# Perioperative outcomes and long-term survival following resection of gallbladder cancer in older adults: retrospective study

**DOI:** 10.1093/bjsopen/zrag052

**Published:** 2026-07-31

**Authors:** Anita Balakrishnan, Petros Barmpounakis, Nikolaos Demiris, Bodil Andersson, Alejandro Brañes, Xavier de Aretxabala, Paul Gibbs, Simon J F Harper, Emmanuel L Huguet, Asif Jah, Vasilis Kosmoliaptsis, Javier Lendoire, Siong S Liau, Shishir K Maithel, Jack L Martin, Colin Noel, Raaj K Praseedom, Alejandro Serrablo, Volkan Adsay, the OMEGA Study Investigators, T Abe, M Abu Hilal, M del Mar Achalandabaso Boira, M Adham, M Adam, M Ahmad, B Al-Sarireh, M Albiol, N Alhaboob, A Alseidi, H Ammar, A Anand, B Andersson, P Antonakis, V Araya, S W Ashley, G Atanasov, F Ausania, R Balestri, A Banerjee, S Banerjee, S Banting, G Barauskas, F Bartsch, A Belli, S Beretta, F Berrevoet, R Singh Bhandari, G Blanco Fernandez, L Bolm, M Bonal, E Bozkurt, A Braat, L Bradshaw, K Bramis, A Branes, L Burdine, M Byrne, M Caceres, M Jesus Castro Santiago, B Chan, L Chong, A Çoker, M Conde Rodriguez, D Croagh, A Crutchley, C Cutolo, M D’Hondt, D D’Souza, F Daams, R Dalla Valle, J Davide, M de Bellis, M de Boer, C de Meyere, P de Reuver, M Dixon, P Dorovinis, G Echeverría Bauer, M Eduarda, H Eker, J Erdmann, M Erkan, E Felekouras, E Felli, E Fernandes, E Figueroa Rivera, A Fulop, D Galun, M Gerhards, P Ghorbani, F Giannone, L Gil, E Giorgakis, M Giuffrida, F Giuliante, I Gkekas, M Gomez Bravo, B Groot Koerkamp, O Guevara, A Guglielmi, A Gulla, R Gupta, A Gupta, M Gutiérrez, A Bakar Hafeez Bhatti, J Hagendoorn, Z Hajee, A R Hakeem, H Hamid, S Hassen, S Heinrich, R Hernandez-Alejandro, R Higuchi, D Hoffman, D Holroyd, D Hughes, A Ivanecz, S Iype, I Jaen Torrejimeno, S Joglekar, R Jones, K Kaczirek, H Kanhere, A Kausar, Z Kee, J Keilson, J Kleef, J Klose, B Knowles, J Kit Koong, N Kumar, S Kunnuru, P Joshi Lakhey, A Laurenzi, Y Sing Lee, F Leon, V Meng Leow, J Lequeu, M Lesurtel, E Lo, S Löb, E Lockie, P Lodge, D López Garnica, V Lopez Lopez, L Lundgren, N Machairas, D Maharjan, D Malde, M Mankarious, G Martel, J Martin, M Mazzola, A Mehrabi, R Memeo, F Milana, G Molina, L Monette, H Morgul, D Moris, A Morsi-Yeroyannis, N Mowbray, F Mulita, E Maria Muttillo, M Nandasena, P Rashid Nashidengo, A Nickkholgh, C Byron Noel, M Ohtsuka, A Ozolins, S Pandanaboyana, N Pararas, A Parente, J Peng, A Perfecto Valero, J Perinel, K Perivoliotis, T Perra, P Pessaux, N Petruch, G Piccolo, L Piros, A Porcu, V Prabakaran, R Prasad, M Prieto Calvo, F Primavesi, E Maria Pueyo Periz, A Quaglia, J Ramia Angel, A Rammohan, F Razionale, R Robles Campos, M Roy, S Rozwadowski, L Ruffolo, N Ruiz, A Ruzzenante, L Saadat, M Amine Said, E Saladino, G Saliba, P Sandstrom, C Alberto Schena, A Scholer, C Schwarz, L Serafini, L Serrablo, P Serrano, D Sharma, A Sheen, V Siddagangaiah, M Silva, S Singh, A Siriwardena, M Skalski, M Smig, F Soliman, A Arun Sonkar, D Sousa Silva, E Sparrelid, H Spiers, P Srinivasan, M Sternby Eilard, O Strobel, U Stupan, M Angel Suarez-Munoz, M Subramaniam, T Sugiura, R Sutcliffe, H Swank, S Talukder, L Taylor, P Bikram Thapa, C Teh, A Thepbunchonchai, C Thieu, N Tiwari, G Torzilli, C Tovikkai, B Trotovsek, S Tsaramanidis, G Tsoulfas, K Uesaka, G Umar, L Urbani, M Vailas, R van Dam, P van de Boezem, S van Laarhoven, T Vanagas, M Van Dooren, M Viennet, L Vigano, A Vijayashanker, C Villodre, T Wakai, A Workneh, L Xu, M Yamamoto, Z Yang, R Young, M Zivanovic

**Affiliations:** Cambridge HPB Unit, Cambridge University Hospitals NHS Foundation Trust, Cambridge, UK; Department of Surgery, University of Cambridge, Cambridge, UK; Cambridge Clinical Trials Unit—Cancer Theme, Cambridge University Hospitals NHS Foundation Trust, Cambridge, UK; Cambridge Clinical Trials Unit—Cancer Theme, Cambridge University Hospitals NHS Foundation Trust, Cambridge, UK; Department of Surgery, Lund University, Skane University Hospital, Lund, Sweden; Department of HPB Surgery, Hospital Dr Sotero del Rio, Santiago, Chile; Department of Digestive Surgery, Hepato-Pancreato-Biliary Surgery Unit, Surgery Service, Gallbladder Consortium Chile, Sotero del Rio Hospital and Clinica Alemana, Santiago, Chile; Cambridge HPB Unit, Cambridge University Hospitals NHS Foundation Trust, Cambridge, UK; Department of Surgery, University of Cambridge, Cambridge, UK; Cambridge HPB Unit, Cambridge University Hospitals NHS Foundation Trust, Cambridge, UK; Department of Surgery, University of Cambridge, Cambridge, UK; Cambridge HPB Unit, Cambridge University Hospitals NHS Foundation Trust, Cambridge, UK; Department of Surgery, University of Cambridge, Cambridge, UK; Cambridge HPB Unit, Cambridge University Hospitals NHS Foundation Trust, Cambridge, UK; Department of Surgery, University of Cambridge, Cambridge, UK; Cambridge HPB Unit, Cambridge University Hospitals NHS Foundation Trust, Cambridge, UK; Department of Surgery, University of Cambridge, Cambridge, UK; Department of Surgery, University of Buenos Aires, Hospital Dr Cosme Argerich, Buenos Aires, Argentina; Cambridge HPB Unit, Cambridge University Hospitals NHS Foundation Trust, Cambridge, UK; Department of Surgery, University of Cambridge, Cambridge, UK; Division of Surgical Oncology, Robert H. Lurie Comprehensive Cancer Centre, Northwestern University, Chicago, Illinois, USA; Cambridge HPB Unit, Cambridge University Hospitals NHS Foundation Trust, Cambridge, UK; Department of Surgery, University of Cambridge, Cambridge, UK; Gastrointestinal Surgery and HPB Surgery, Department of Surgery, University of the Free State, Bloemfontein, South Africa; Cambridge HPB Unit, Cambridge University Hospitals NHS Foundation Trust, Cambridge, UK; Department of Surgery, University of Cambridge, Cambridge, UK; Department of HPB Surgery, Miguel Servet University Hospital, Zaragoza, Spain; Department of Pathology, Koç University Hospital, Istanbul, Turkey; Koç University Research Center for Translational Medicine (KUTTAM), Istanbul, Turkey

**Keywords:** older adults, gallbladder cancer, surgery, cholangiocarcinoma

## Abstract

**Background:**

Gallbladder cancer (GBC) is a rare but aggressive disease, and surgical resection remains the only potential curative treatment. Although tumour-related effects on prognosis are well established, the impact of age is less understood. This study aimed to evaluate the influence of age on overall survival (OS), recurrence-free survival (RFS), and perioperative complications in GBC.

**Methods:**

Data from patients undergoing curative resection for GBC at 133 centres across 41 countries between 2010 and 2020 were analysed to determine the prognostic association of age ≥ 75 years with OS, RFS, and morbidity. Propensity score matching was used to address confounders between the two age groups.

**Results:**

In all, 4138 patients underwent surgery for GBC. Patients with macroscopic tumour remaining after surgery, metastatic disease, only high-grade dysplasia were excluded leaving 3676 patients for analyses. Full data on all relevant parameters was available for 2072 patients aged < 75 years and 633 patients aged ≥ 75 years. Patients aged ≥ 75 years had more co-morbidities, underwent less extensive surgery or lymphadenectomy, and received adjuvant chemotherapy less frequently than younger (< 75 years) patients. Age ≥ 75 years was associated with poorer OS in both the unmatched (hazard ratio (HR) 1.34; 95% confidence interval (c.i.) 1.14 to 1.56; *P* < 0.001) and matched cohorts (HR 1.31; 95% c.i. 1.12 to 1.54; *P* < 0.001) cohorts, but was not associated with RFS or 1-year survival. Tumour extent and nodal stage had the greatest association with OS and RFS. Age was not associated with increased complications in either the unmatched (odds ratio (OR) 1.11; 95% c.i. 0.85 to 1.45; *P* = 0.400) or matched (OR 0.90; 95% c.i. 0.72 to 1.12; *P* = 0.353) cohorts.

**Conclusions:**

Older adults received less extensive surgery and infrequent adjuvant chemotherapy. Age ≥ 75 years was associated with poorer OS following GBC resection but comparable complication rates to younger adults. Older adults of sufficient fitness should not be denied curative treatment based on age, and oncological benefit should be balanced against perioperative risk to personalize treatment and optimize surgical outcomes.

## Teaser

This largest study of the surgical outcomes of gallbladder cancer to date shows that older (age ≥ 75 years) patients underwent less aggressive surgery, less lymphadenectomy, and less chemotherapy than younger patients. Older patients had poorer overall survival but similar recurrence-free survival and complication rates compared with younger patients in propensity score-matched cohorts. The data suggest that age alone should not be a barrier to the contemplation of curative surgery for gallbladder cancer in fit older patients, and that these patients should be considered for newer targeted treatments and immunotherapy to prolong survival.

## Introduction

Gallbladder cancer (GBC) is a rare but notably aggressive malignancy, with highest incidence in parts of East Asia and South America^1^. Although surgical resection remains the only potential curative modality, management strategies for GBC vary widely, from simple cholecystectomy for early-stage disease to extended resections involving major hepatectomy, extrahepatic bile duct resection, and the resection of adjacent organs, with inconsistent survival benefits and substantial perioperative risks^2^.

Although tumour-centric characteristics such as T and N category, lymphovascular invasion, perineural invasion, and differentiation are established prognostic factors in GBC, less attention has been paid to patient-level determinants, particularly age at the time of surgery. This is in stark contrast to hepatocellular and pancreatic cancer, where age has been more extensively studied as a variable influencing postoperative and long-term outcomes.

A recent Surveillance, Epidemiology, and End Results Medicare-based analysis^3^ suggested that older adults may not always receive curative approaches to operable GBC, whereas Patkar *et al*.^4^ demonstrated an association between advancing age and worse overall survival (OS) in a mixed cohort that included resected and metastatic GBC.

To date there have been no large multicentre studies specifically evaluating the effect of age on surgical outcomes for GBC in appropriately matched cohorts. Furthermore, few studies have evaluated how age interacts with other prognostic factors, such as resection extent, tumour biology, or the use of adjuvant therapy, to influence both short-term and long-term outcomes. Understanding these relationships is especially critical in an ageing global population, where surgical decision-making must increasingly balance oncological benefit with physiological reserve and postoperative risk.

The primary aim of the present study was to assess the association between age and OS and recurrence-free survival (RFS). The secondary aim was to evaluate the association between age and postoperative complications.

## Methods

### Data collection

Collaborating centres were recruited by invitation disseminated via e-mail to all members of the three international hepatopancreatobiliary (HPB) associations (the European–African HPB Association, the Americas HPB Association, and the Asia-Pacific HPB Association).Patients who had undergone surgery for GBC diagnosed before surgery or incidentally (for example, identified following cholecystectomy for benign disease) were included in this study. Histological staging was classified according to the eighth American Joint Committee on Cancer classification (2018) for GBC^5^. Exclusion criteria were high-grade dysplasia with no invasive disease, metastatic disease at the time of surgery, or macroscopic tumour remaining at the end of resection (R2 resections).

#### Demographic and pathological data collection

Clinical parameters, operative details, pathological findings, and follow-up and survival data were obtained from institutional databases. Co-morbidities were used to calculate the Charlson co-morbidity index (CCI)^6^. Countries were classified according to the 2021 World Bank categories as high-income countries and upper-middle, lower-middle, or low income countries, with the latter three groups amalgamated to a single category of low- or middle-income countries (LMICs)^7^.

#### Operative details

The extent of surgery was defined as cholecystectomy only (CO), liver resection (LR; either a wedge resection taking a margin of the liver at the gallbladder bed or resection of liver segments IVb and V), or major resection (MR; incorporating any of the following: resection of four or more segments of the liver, excision of the extrahepatic bile duct, vascular or additional organ resection). Complications were graded according to the Clavien–Dindo (CD) classification^8^, with the rate of 30-day CD ≥ IIIa complications analysed. Classifications of liver-specific postoperative complications had not been in uniform use for most of the time frame of this study, and were not retrospectively applied to minimize bias.

#### Follow-up and survival

OS was defined as the interval between the date of surgery for GBC (for patients who underwent further surgery for incidental GBC, the date of the second operation was used) and the date of death, obtained from hospital or government records at the time of study closure. RFS was defined as the time interval between the date of surgery and either the date of first identification of recurrence on imaging or histology or the date of most recent imaging excluding recurrence.

### Ethics approval

Institutional and ethics approval for this study was obtained from the Research and Development Office at Cambridge University Hospitals NHS Foundation Trust (the lead site) and the UK Research Ethics Committee (IRAS ID 285918). Informed consent from patients was deemed unnecessary by the ethics committee for this retrospective study. Other participating centres obtained further institutional and national approvals as needed. This study was conducted and is reported in compliance with the STROBE guidelines for cohort studies^9^.

### Statistical analysis

Median follow-up was calculated from the Kaplan–Meier estimate and the associated 95% confidence interval (c.i.). Prognostic parameters for survival and complication rates were identified from the literature. Associations between these parameters and outcome variables were assessed using Cox proportional hazards multivariable regression models, with all relevant assumptions met. Cox regression analysis was used for OS and RFS and logistic regression analysis was used for 1-year survival and complications (taken as CD ≥ IIIA). Subgroup univariable analyses were performed to assess the effects of age on survival and complication rates. The significance of differences between categorical variables on subgroup analyses was assessed using the χ^2^ test and Fisher’s exact test.

Confounding by age was corrected by performing covariate matching on age groups using three distinct strategies: full matching, optimal matching, and nearest-neighbour matching. Age was the primary confounder of interest, and the matching aimed to align the distribution of age groups between exposure groups for variables such as sex, margin status, T category, nodal stage, extent of surgery, adjuvant chemotherapy and radiotherapy, country income level and co-morbidities (see *supplementary methods*).

In this cohort of patients, the nearest-neighbour matching technique yielded the smallest standard mean differences (all < 0.1), indicating the greatest degree of matching, and was thus used as the default technique for analysis of the results. As a sensitivity measure, analysis of the optimal matching technique findings was also performed. All statistical analyses were conducted in R v4.5.1 (2025; R Foundation for Statistical Computing, Vienna, Austria).

## Results

### Demographic differences by age bracket

In all, 4138 patients were identified who had undergone surgery for GBC at 133 participating centres in 41 countries between 1 January 2010 and 31 December 2020 (*Fig. S1*). Of these, 384 with macroscopic tumour remaining after surgery or metastatic disease and 78 with only high-grade dysplasia were excluded, leaving a final total of 3676 patients for analysis. The median age at time of surgery was 66 (interquartile range (i.q.r.) 58–74) years. A cut-off of 75 years was chosen to define older adults based on clinically relevant numbers of patients in each of the two resulting groups in this cohort, upper limits for surgical intervention across both high- and low-income countries, and cut-offs in the current literature for other tumour types. This cut-off yielded 1445 patients aged ≥ 75 years. OS and RFS were available for 3622 and 2991 patients, respectively. The median follow-up duration was 45.3 (i.q.r. 24.1–80.5) months. Median OS and RFS for the full cohort were 51.2 (i.q.r. 49.3–52.8) and 35.2 (i.q.r. 34.3–36.9) months, respectively.

Full data on all relevant parameters was available for 2072 patients aged < 75 years and 633 patients aged ≥ 75 years (*Table S2*). Only 6.3% (40) of all patients aged ≥ 75 years undergoing curative GBC surgery were from non-high-income countries, compared with 29.4% (609) of patients in the younger cohort. Co-morbidities were more prevalent in the older group, with a CCI ≥ 5 observed in 426 patients (67.3%), compared with 586 patients (28.3%) in the younger group. Older adults were also less likely to undergo extensive surgery; 124 (19.6%) underwent CO, compared with 168 younger adults (8.1%; *P* < 0.001; *Table 1*). Similarly, lymphadenectomy was performed less frequently in patients aged ≥ 75 years, with 113 patients recorded as Nx, compared with 172 in younger cohort (*P* < 0.001). Older adults had fewer lymph nodes excised (median 3; range 0–53) than younger adults (median 6; range 0–68), and a smaller proportion of older adults met the recommended lymph node yield of ≥ 6 (306 *versus* 134 patients). Adjuvant chemotherapy was also less commonly administered to the older than younger age group (126 *versus* 927 patients; *P* < 0.001).

**Table 1:** Demographic data comparing patients aged < 75 and ≥ 75 years, with *P* before and after propensity score matching with the nearest-neighbour technique

	Age < 75 years	Age ≥ 75 years	*P*		SMD
			Before PSM	After PSM	
All patients (*n* = 2705)	2072 (76.6%)	633 (23.4%)			
**Sex**					
Male (*n* = 957)	709 (34.2%)	248 (39.2%)	0.025	0.954	0.007
Female (*n* = 1748)	1363 (65.8%)	385 (60.8%)			
**Charlson co-morbidity index**					
< 5 (*n* = 1693)	1486 (71.7%)	207 (32.7%)	< 0.001	0.905	0.010
≥ 5 (*n* = 1012)	586 (28.3%)	426 (67.3%)			
**T category**					
T1a (*n* = 131)	101 (4.9%)	30 (4.7%)	0.011	0.410	0.112
T1b (*n* = 298)	230 (11.1%)	68 (10.7%)			0.010
T2 (*n* = 1270)	940 (45.4%)	330 (52.1%)			0.063
T3 (*n* = 856)	673 (32.5%)	183 (28.9%)			0.021
T4 (*n* = 150)	128 (6.2%)	22 (3.5%)			0.026
**N category**					
N0 (*n* = 1427)	1111 (53.6%)	316 (49.9%)	< 0.001	0.375	0.047
N1 (*n* = 785)	618 (29.8%)	167 (26.4%)			0.021
N2 (*n* = 208)	171 (8.3%)	37 (5.8%)			0.013
Nx (*n* = 285)	172 (8.3%)	113 (17.9%)			0.094
**Residual tumour classification**					
R0 (*n* = 2397)	1837 (88.7%)	560 (88.5%)	0.952	0.929	0.010
R1 (*n* = 308)	235 (11.3%)	73 (11.5%)			
**Extent of resection**					
CO (*n* = 292)	168 (8.1%)	124 (19.6%)	< 0.001	0.302	0.084
LR (*n* = 1572)	1243 (60.0%)	329 (52.0%)			0.035
MR (*n* = 841)	661 (31.9%)	180 (28.4%)			0.035
**Adjuvant chemotherapy**					
Yes (*n* = 1053)	927 (44.7%)	126 (19.9%)	< 0.001	0.067	0.111
No (*n* = 1652)	1145 (55.3%)	507 (80.1%)			
**Adjuvant radiotherapy**					
Yes (*n* = 132)	120 (5.8%)	12 (1.9%)	< 0.001	1.000	0.012
No (*n* = 2573)	1952 (94.2%)	621 (98.1%)			
High-income country (*n* = 2056)	1463 (70.6%)	593 (93.7%)	< 0.001	1.000	0.006
Non-high-income country (*n* = 649)	609 (29.4%)	40 (6.3%)			

Values are *n* (%). SMD, standard mean difference; PSM, propensity score matching; CO, cholecystectomy only; LR, limited liver resection; MR, major resection.

### OS analysis

**Fig. 1:**
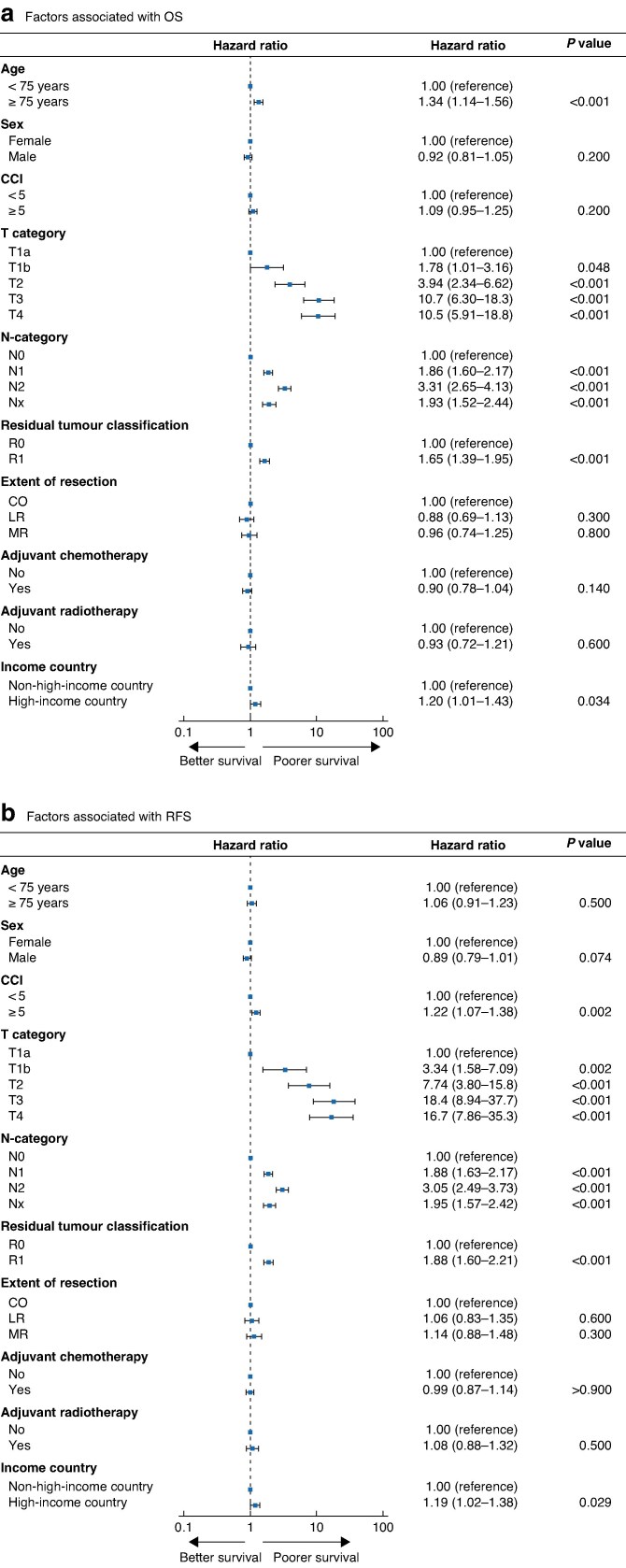
Forest plot showing multivariable Cox regression analysis of factors associated with OS and RFS in the full cohort **a** OS and **b** RFS. Values in parentheses and error bars show 95% confidence intervals. OS, overall survival; RFS, recurrence-free survival; CCI, Charlson co-morbidity index; CO, cholecystectomy only; LR, limited liver resection; MR, major resection.

**Fig. 2:**
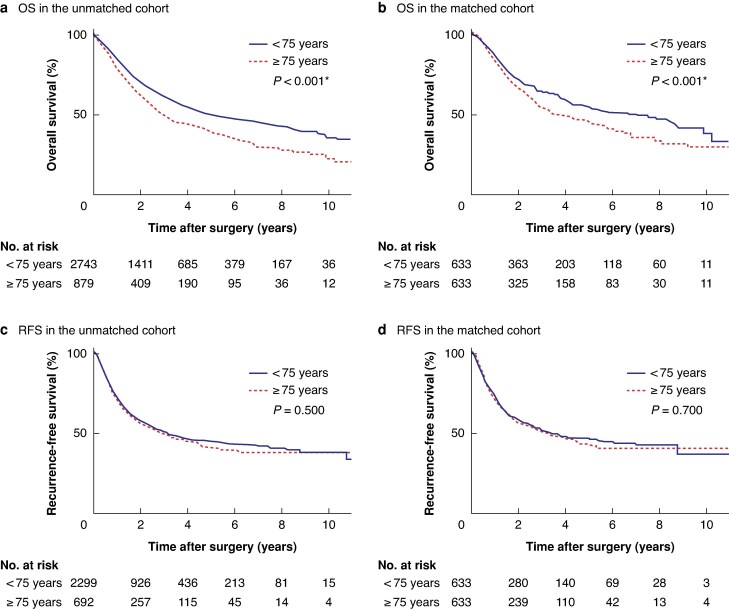
Kaplan–Meier curves showing OS and RFS in the unmatched and matched cohorts **a** OS in the unmatched cohort, **b** OS in the matched cohort, **c** RFS in the unmatched cohort, and **d** RFS in the matched cohort. OS, overall survival; RFS, recurrence-free survival.

In the unmatched cohort of patients, the older age group was associated with a statistically significant 34.0% higher risk of death (95% c.i. 1.14 to 1.56, *P* < 0.001) from any cause on multivariable analysis, with a significantly lower 5-year OS compared with younger patients (38.5 *versus* 49.3%; *P* < 0.001 on univariable analysis; *Figs 1a* and *2a*). Increasing T category showed the greatest association with poorer survival (hazard ratio (HR) 10.5; 95% c.i. 5.91 to 18.8; *P* < 0.001 for T4 *versus* T1a disease). N category, positive resection margins, and treatment in a high-income country were also associated with poorer OS. Age demonstrated a small interaction with income for OS (HR 0.98; 95% c.i. 0.97 to 1.00; *P* = 0.032; *Fig. S2a*). Sex, CCI ≥ 5, receipt of chemotherapy or radiotherapy, and the extent of surgical resection showed no significant association with OS.

Nearest-neighbour propensity score matching showed a statistically significant 31% higher risk of all-cause death for patients in the older age group (95% c.i. 1.12 to 1.54; *P* < 0.001; *Fig. 2b*). Sensitivity analysis of OS using only preoperative variables in the matched cohort showed similar findings (HR 1.31; 95% c.i. 1.11 to 1.55; *P* < 0.002). Ninety-day and 120-day landmark analyses confirmed a similar association between the older age group and poorer OS as seen in the original analysis (*Fig. S3a,b*). This association between older age and poorer OS remained consistent even after exclusion of patients with Nx status (both matched and unmatched cohorts; *Fig. S4a*). Age exhibited a significant non-linear association with OS, with almost exponentially higher risk for the oldest adults, and increasing risk to OS with progressively higher age brackets (*Figs S5 and S6a,b*).

### RFS analysis

There was no significant difference in 5-year survival for patients aged ≥ 75 years on either multivariable (HR 1.06; 95% c.i. 0.91 to 1.23; *P* = 0.500, *Fig. 1b*) or univariable (40.8 *versus* 43.9% in younger patients; *P* = 0.356; *Fig. 2c*) analysis compared with younger patients. Poorer RFS was associated with T category, N category, margin status, CCI ≥ 5, and having surgery in a high-income country (*Fig. 1b*). Age showed a small interaction with country income level for RFS (HR 0.98; 95% c.i. 0.97 to 0.99; *P* = 0.004; *Fig. S2b*). There was no association between age group and RFS in the matched cohort (HR 1.03; 95% c.i. 0.89 to 1.21; *P* = 0.700; *Fig. 2d*). Sensitivity analysis of RFS using only preoperative variables in the matched cohort showed similar findings (HR 1.05; 95% c.i. 0.89 to 1.23; *P* = 0.600). Ninety-day and 120-day landmark analyses also showed no association between older age and RFS (*Fig. S7a,b*). The lack of association between the older age group and RFS remained consistent in both the matched and unmatched patients after excluding patients with Nx status from the analysis (*Fig. S4b*). There was a (likely clinically insignificant) non-linear risk associated with RFS when age was assessed as a continuous variable, but no associated risk to RFS with age cut-offs of > 70 or > 80 years (*Figs S8 and S9a,b*).

### Survival at 1 year

The older age group had a 25% reduction in likelihood of surviving 1 year after surgery (*P* = 0.061). Poorer 1-year OS was associated with increasing T category (odds ratio (OR) 0.06 (95% c.i. 0.03 to 0.18) for T4 *versus* T1a; *P* < 0.001) and N category (OR 0.28 (95% c.i. 0.19 to 0.42) for N2 *versus* N0; *P* < 0.001). Greater co-morbidity (CCI ≥ 5) was associated with a non-significant 21% decrease in 1-year survival (95% c.i. 0.61 to 1.04; *P* = 0.089). The receipt of adjuvant chemotherapy was associated with better 1-year survival (OR 1.74; 95% c.i. 1.31 to 2.31; *P* < 0.001; *Fig. S10*). In the matched cohorts, the older age group had a small, non-significant association with poorer 1-year survival (OR 0.96; 95% c.i. 0.92 to 1.01; *P* = 0.097).

### Risk factors for complications

**Fig. 3:**
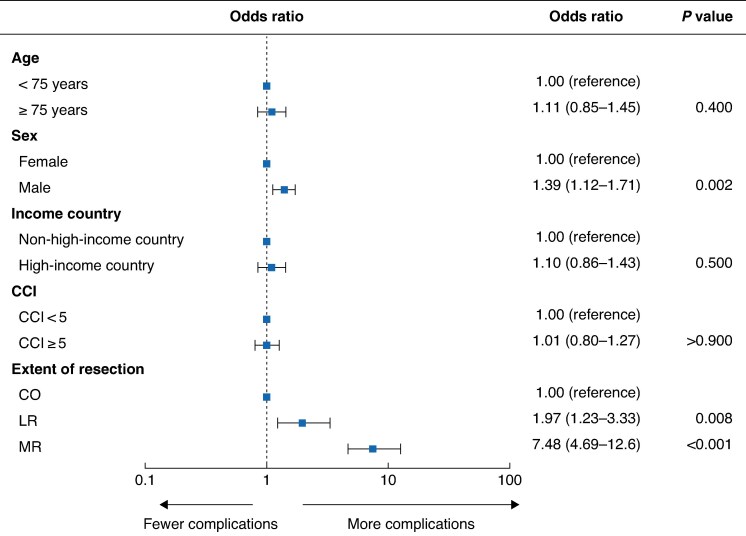
Forest plot showing multivariable logistic regression of factors associated with postoperative complications Values in parentheses and error bars show 95% confidence intervals. CCI, Charlson co-morbidity index; CO, cholecystectomy only; LR, limited liver resection; MR, major resection.

Age ≥ 75 years was associated with no additional associated risk of complications compared with younger age in either multivariable analysis (OR 1.11; 95% c.i. 0.85 to 1.45; *P* = 0.400) or univariable analysis (20.0 *versus* 18.9% compared with younger age; *P* = 0.457). Increasing the extent of surgery was associated with the greatest risk of complications, with ORs of 7.47 (95% c.i. 4.69 to 12.60) for MR and 1.97 (95% c.i. 1.23 to 3.33) for limited LR *versus* CO (*Fig. 3*). Male sex was also associated with a greater risk of complications (OR 1.39; 95% c.i. 1.12 to 1.71; *P* = 0.002). After matching for sex, country income level, CCI, and the extent of surgery, older age was no longer associated with a significant increase in complications compared with age < 75 years (OR 0.90; 95% c.i. 0.72 to 1.12; *P* = 0.353).

### Prognostic factors in the older age group

**Fig. 4:**
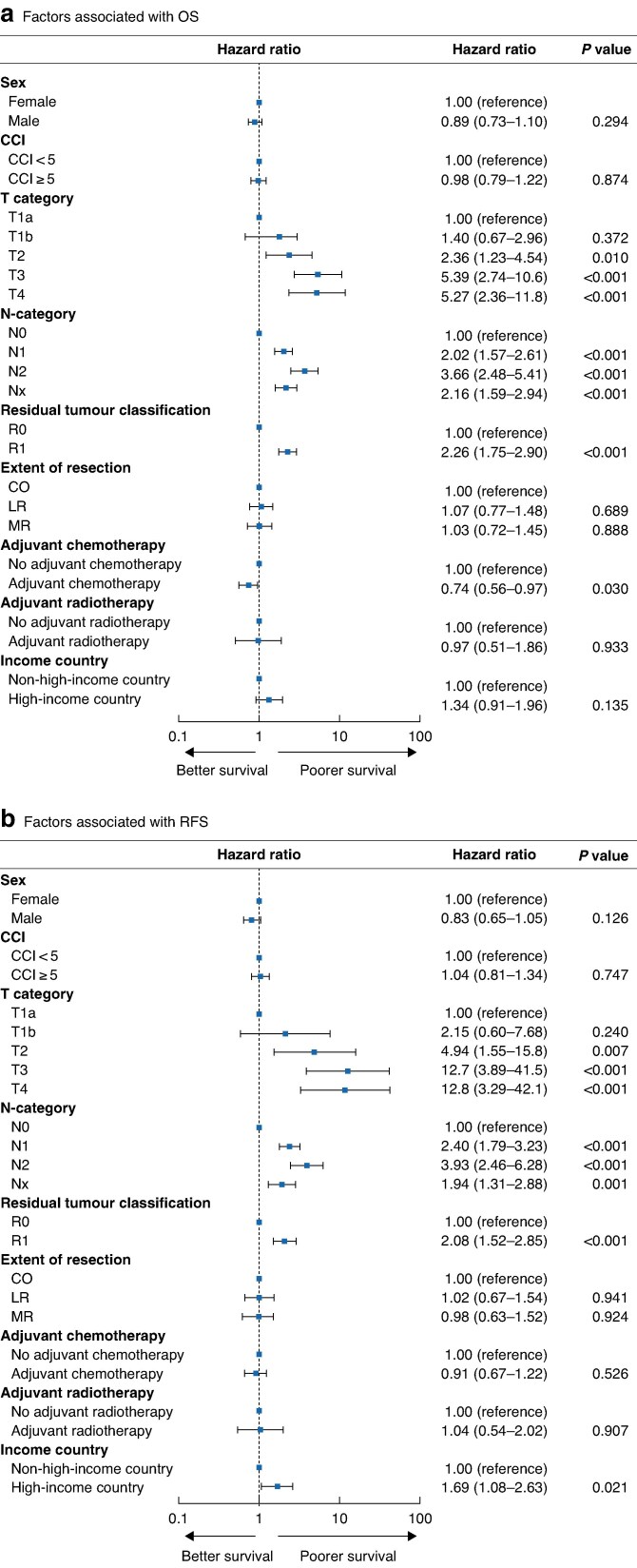
Forest plot showing multivariable Cox regression of factors associated with OS and RFS according to age ≥ 75 and <75 years **a** OS and **b** RFS. Values in parentheses and error bars show 95% confidence intervals. OS, overall survival; RFS, recurrence-free survival; CCI, Charlson co-morbidity index; CO, cholecystectomy only; LR, limited liver resection; MR, major resection.

On analysis of the ≥ 75 year cohort alone, increased T category (HR 5.27 (95% c.i. 2.36 to 11.75) for T4 *versus* T1a; *P* < 0.001), increased N category (HR 3.66 (95% c.i. 2.48 to 5.41) for N2 *versus* N0; *P* < 0.001), and positive margins (HR 2.26; 95% c.i. 1.75 to 2.90; *P* < 0.001) were associated with poorer OS (*Fig. 4a*). The same prognostic factors were also associated with RFS and 1-year survival (*Fig. 4b* and *Fig. S11*). The receipt of adjuvant chemotherapy was associated with improved OS (HR 0.74; 95% c.i. 0.56 to 0.97; *P* = 0.0299) and 1-year survival (OR 2.01; 95% c.i. 1.29 to 3.42; *P* = 0.009), but not RFS (HR 0.91; 95% c.i. 0.67 to 1.22; *P* = 0.526). In contrast, adjuvant chemotherapy had no significant association with better OS in the 90-day and 120-day landmark analyses (HR 0.98 (95% c.i. 1.71 to 1.36; *P* = 0.910) and 0.88 (95% c.i. 0.61 to 1.27; *P* = 0.521), respectively; *Fig. S12a,b*). Older adults had a higher rate of complications in association with extended resections (OR 3.09; 95% c.i. 1.86 to 5.14; *P* < 0.001; *Fig. S13*). Having more co-morbidities (CCI ≥ 5) was associated with a 45% increase in complications compared with CCI < 5 (OR 1.45; 95% c.i. 0.98 to 2.14; *P* = 0.063). Thirty-five patients in the ≥ 75-year cohort died within 90 days of surgery (*Table S2*). Sepsis (primarily related to bile leak) was the most common cause of death in most older adults (13 patients), whereas liver failure was the primary cause of death in younger adults (27 patients).

## Discussion

In this large multicentre cohort study of the outcomes from curative resection for GBC, older (≥ 75 years) age was associated with poorer OS than younger age, but RFS, 1-year survival, and complication rates were comparable between the two groups.

As reported for other gastrointestinal cancers^10–12^, older adults in the present study underwent more extensive surgery or lymphadenectomy less frequently. They were also less often from LMICs, possibly related to delayed presentation, treatment costs, access to tertiary surgical and perioperative care, and patient expectations and willingness for major surgery. Geriatric oncology in LMICs is an important research focus, given the predicted increase in cancers in their older populations in the coming two decades^13^.

Only a minority of older adults received adjuvant treatment, comparable to data from pancreatic and colorectal malignancies^14,15^. The reasons are probably related to pre-existing or postoperative co-morbidities, or delayed postoperative recovery. Previous studies^16,17^ showed similar outcomes from adjuvant chemotherapy for older and younger adults, recommending more consistent consideration of chemotherapy in fit older adults to maximize the survival benefit from curative resection. Newer immunotherapy and targeted treatments with less toxicity may also prove beneficial for older adults^18–21^.

An association between age and reduced OS, but not RFS, was noted in the present study. These findings may represent a higher baseline risk of death from non-cancer causes in older adults, such as cardiovascular disease, infections or new co-morbidities or other frailty-related complications. Although time to recurrence may have been similar between older and younger cohorts, OS in older adults may have been disproportionately influenced by less aggressive treatment of recurrence. Those who received adjuvant or palliative chemotherapy may have been more prone to dose reductions or early discontinuation to prioritize quality of life. Disease stage or the extent of surgery were also strongly associated with prognosis in the present study, in keeping with data for other gastrointestinal cancers^22,23^. Non-cancer causes of death were not available for most patients in this study, and it was difficult to delineate tumour-related from age-related mortality.

Previous studies have yielded conflicting data on the additional risk of postoperative complications from HPB resections associated with increasing age^10,14,24–26^. The present study showed no difference in the postoperative complication rate between older and younger adults, or in 1-year survival, a longer-term marker for postoperative complications^27^. One explanation, as seen in data from patients with colorectal liver metastases, may be that older adults underwent proportionally less extensive surgery, such as pancreaticoduodenectomies^14,25,28^. Older adults in the present study had no increase in complication rates even for matched extents of surgery. These findings therefore more likely indicate appropriate patient selection, avoiding extensive surgery in high co-morbidity patients. This suggests that older adults could be considered for all curative treatments, including major resections, if sufficiently fit, without necessarily incurring an increase in postoperative complications^29^. Careful preoperative assessment of older adults, including standardized frailty indices, should form an essential component of perioperative guidelines internationally. Perioperative geriatrician input may be particularly useful for complex older adults. The 1-year OS was noted to be better in high-income countries, possibly reflecting better rescue of delayed postoperative complications.

The main strength of this study lies in the inclusion of a very large number of centres globally, incorporating high- and low-income countries, as well as regions with a low and high incidence of GBC. Despite this, there are some inevitable limitations related to the retrospective nature of the study. The CCI allows objective and quantifiable assessment of co-morbidities, but information on condition severity, or the degree of frailty or disability from each co-morbidity, was generally unavailable. It was also not possible to assess more nuanced effects of surgical intervention, including postoperative loss of independence or failure to return to preoperative physical and cognitive function. There was no internationally uniformly applied frailty index at the time of this study. In addition, many patients were unfortunately no longer alive at the time of the study for quality of life parameters to be assessed. Adjuvant chemotherapy details were not available for most patients, thus biases introduced by selection cannot be excluded, such as immortal-time and centre practice heterogeneity. Because the older adults included in this study were a subpopulation considered fit for major surgery, comparisons with older adults not undergoing surgery were not undertaken, as these would have been inevitably confounded by higher levels of co-morbidities.

This study shows that older adults receive less extensive resections and lymphadenectomy than younger patients, as well as less adjuvant chemotherapy. Although OS is poorer, postoperative complication rates are comparable to those in younger patients. Curative treatment options, including adjuvant chemotherapy, should therefore be considered in sufficiently fit older adults regardless of age. These data provide an important overview of the current management of GBC in older adults worldwide, and highlight the need for more individualized treatment strategies balancing oncological benefit against perioperative risk to optimize surgical outcomes.

## Collaborators

The members of the OMEGA Study Investigators are: T. Abe (Onomichi General Hospital, Hiroshima, Japan); M. Abu Hilal (Poliambulanza Foundation Hospital, Brescia, Italy); M. del Mar Achalandabaso Boira (Hospital Universitario Vall d´Hebron, Barcelona, Spain); M. Adham (Edouard Herriot Hospital, Lyon, France); M. Adam (University of California San Francisco, California, USA); M. Ahmad (Emory University, Atlanta, Georgia, USA); B. Al-Sarireh (Morriston Hospital, Swansea, UK);M. Albiol (Hospital Universitari Girona Dr Josep Trueta, Girona, Spain); N. Alhaboob (Soba University Hospital, Khartoum, Sudan); A. Alseidi (University of California San Francisco, California, USA); H. Ammar (Sahloul Hospital, Tunisia); A. Anand (King George’s Medical University, Lucknow, India); B. Andersson (Skane University Hospital, Lund, Sweden); P. Antonakis (Aretaieion University Hospital, Athens, Greece);V. Araya (Hospital Sotero del Rio and Pontificia Catolica Universidad de Chile, Santiago, Chile); S. W. Ashley (Brigham and Women’s Hospital, Boston, USA); G. Atanasov (Royal Adelaide Hospital, Adelaide, Australia); F. Ausania (Hospital Clinic Barcelona, Barcelona, Spain); R. Balestri (Azienda Ospedaliero Universitaria Pisana, Pisa, Italy); A. Banerjee (Royal Free Hospital, London, United Kingdom); S. Banerjee (Tata Medical Centre, Kolkata, India); S. Banting (Melbourne Surgical Group, Melbourne, Australia); G. Barauskas (Hospital of Lithuanian University of Health Sciences Kauno Klinikos, Kaunas, Lithuania); F. Bartsch (Universitätsmedizin Mainz, Mainz, Germany); A. Belli (National Cancer Institute, G Pascale, Napoli, Italy); S. Beretta (ASST Grande Ospedale Metropolitano Niguarda, Italy); F. Berrevoet (University Hospital Ghent, Ghent, Belgium); R. Singh Bhandari (Tribhuvan University Teaching Hospital, Kathmandu, Nepal); G. Blanco Fernandez (University of Extremadura, Badajoz, Spain); L. Bolm (University Medical Center, Lübeck, Germany); M. Bonal (Croix Rousse University Hospital , Lyon, France); E. Bozkurt (Koc University Hospital, Istanbul, Turkey); A. Braat (Leiden University Medical Center, Leiden, Netherlands); L. Bradshaw (St Vincent’s Hospital, Melbourne, Australia); K. Bramis (Aretaieion University Hospital, Athens, Greece); A. Branes (Hospital Sotero del Rio and Pontificia Catolica Universidad de Chile, Santiago, Chile); L. Burdine (University of Arkansas for Medical Sciences, Arkansas, USA); M. Byrne (University of Rochester Medical College, New York, USA); M. Caceres (Dos de Mayo Hospital, Lima, Peru); M. Jesus Castro Santiago (Hospital Universitario Puerta del Mar, Cadiz, Spain); B. Chan (Royal Liverpool University Hospital, Liverpool, United Kingdom); L. Chong (St Vincent’s Hospital, Melbourne, Australia); A. Çoker (Izmir HPB Clinic, Izmir, Turkey); M. Conde Rodriguez (Hospital Universitario Lucus Augusti, Lugo, Spain); D. Croagh (Monash Hospital, Melbourne, Australia); A. Crutchley (Leeds Teaching Hospitals NHS Trust, Leeds, UK); C. Cutolo (National Cancer Instititute G Pascale, Napoli, Italy); M. D’Hondt (AZ Groeninge, Kortrijk, Belgium); D. D’Souza (McMaster University, Hamilton, Ontario, Canada); F. Daams (Amsterdam UMC, Netherlands); R. Dalla Valle (Parma University Hospital, Parma, Italy); J. Davide (Centro Hospitalar Universitário do Porto, Porto, Portugal); M. de Bellis (University of Verona Medical School, Verona, Italy); M. de Boer (University Medical Center Groningen, Netherlands); C. de Meyere (AZ Groeninge, Kortrijk, Belgium); P. de Reuver (Radboud UMC, Nijmegen, Netherlands); M. Dixon (Penn State College of Medicine, Pennsylvania, USA); P. Dorovinis (Laiko General Hospital and Kapodistrian University of Athens, Greece); G. Echeverría Bauer (Clinical Alemana, Santiago, Chile); M. Eduarda (Federal University of Rio de Janeiro, Rio de Janeiro, Brazil); H. Eker (University Hospital Ghent, Ghent, Belgium); J. Erdmann (Amsterdam UMC, Netherlands); M. Erkan (Koc University Hospital, Istanbul, Turkey); E. Felekouras (Laiko General Hospital and Kapodistrian University of Athens, Greece); E. Felli (Nouvel Hospital Civil, Strasbourg, France); E. Fernandes (Federal University of Rio de Janeiro, Rio de Janeiro, Brazil); E. Figueroa Rivera (Hospital Clínico Regional Dr Guillermo Grant Benavente, Concepcion, Chile); A. Fulop (Semmelweis University, Budapest, Hungary); D. Galun (University Clinical Center of Serbia, Belgrade, Serbia); M. Gerhards (OLVG, Amsterdam, Netherlands); P. Ghorbani (Karolinska University Hospital, Stockholm, Sweden); F. Giannone (Nouvel Hospital Civil, Strasbourg, France); L. Gil (University of Buenos Aires, Buenos Aires, Argentina); E. Giorgakis (University of Arkansas for Medical Sciences, Arkansas, USA); M. Giuffrida (Parma University Hospital, Parma, Italy); F. Giuliante (Fondazione Policlinico Universitario A. Gemelli, Rome, Italy); I. Gkekas (Kirurgcentrum, Umea University, Umea, Sweden); M. Gomez Bravo (Hospital Virgen Del Rocio, Spain); B. Groot Koerkamp (Erasmus MC, Rotterdam, Netherlands); O. Guevara (Colombian National University, Bogota, Colombia); A. Guglielmi (University of Verona Medical School, Verona, Italy); A. Gulla (Vilnius University Hospital, Vilnius, Lithuania); R. Gupta (Synergy Institute of Medical Sciences, Dehradun, India); A. Gupta (All India Institute of Medical Sciences, Rishikesh, India); M. Gutiérrez (Hospital Miguel Servet, Zaragoza, Spain); A. Bakar Hafeez Bhatti (Shifa International Hospital, Islamabad, Pakistan); J. Hagendoorn (UMC Utrecht Cancer Center, Utrecht, Netherlands); Z. Hajee (Manchester Foundation NHS Trust, Manchester, UK); A. R. Hakeem (Leeds Teaching Hospitals NHS Trust, Leeds, UK); H. Hamid (Soba University Hospital, Khartoum, Sudan); S. Hassen (Eastern Health Box Hill, Australia); S. Heinrich (Universitätsmedizin Mainz, Mainz, Germany); R. Hernandez-Alejandro (University of Rochester Medical College, New York, USA); R. Higuchi (Tokyo Women’s Medical Hospital, Tokyo, Japan); D. Hoffman (University of California San Francisco, California, USA); D. Holroyd (Glasgow Royal Infirmary, Glasgow, UK); D. Hughes (Oxford University Hospitals NHS Foundation Trust, Oxford, UK); A. Ivanecz (University Medical Center Maribor, Maribor, Slovenia); S. Iype (Royal Free Hospital, London, UK); I. Jaen Torrejimeno (University of Extremadura, Badajoz, Spain); S. Joglekar (Eastern Health Box Hill, Australia); R. Jones (Royal Liverpool University Hospital, Liverpool, UK); K. Kaczirek (University of Medicine Vienna, Austria); H. Kanhere (Royal Adelaide Hospital, Adelaide, Australia); A. Kausar (Royal Blackburn Hospital, Blackburn, UK); Z. Kee (Hospital Sultanah Bahiyah, Alor Setar, Malaysia); J. Keilson (Emory University, Atlanta, Georgia, USA); J. Kleef (University Medicine Halle, Halle, Germany); J. Klose (University Medicine Halle, Halle, Germany); B. Knowles (Royal Melbourne Hospital, Melbourne, Australia); J. Kit Koong (University Malaya Medical Centre, Kuala Lumpur, Malaysia); N. Kumar (University Hospital of Wales, Cardiff, UK); S. Kunnuru (Royal Free Hospital, London, UK); P. Joshi Lakhey (Tribhuvan University Teaching Hospital, Kathmandu, Nepal); A. Laurenzi (University of Bologna, Bologna, Italy); Y. Sing Lee (University Malaya Medical Centre, Kuala Lumpur, Malaysia); F. Leon (Hospital Clinic Barcelona, Barcelona, Spain); V. Meng Leow (Hospital Sultanah Bahiyah, Alor Setar, Malaysia); J. Lequeu (Dijon University Hospital, Dijon, France); M. Lesurtel (Croix Rousse University Hospital, Lyon, France); E. Lo (McMaster University, Hamilton, Ontario, Canada); S. Löb (University Hospital Würzburg, Würzburg, Germany); E. Lockie (Royal Melbourne Hospital, Melbourne, Australia); P. Lodge (Leeds Teaching Hospitals NHS Trust, Leeds, UK); D. López Garnica (Hospital Universitario Vall d´Hebron, Barcelona, Spain); V. Lopez Lopez (Clinic and University Virgen de la Arrixaca Hospital, Murcia, Spain); L. Lundgren (University Hospital Linkoping, Sweden); N. Machairas (Laiko General Hospital and Kapodistrian University of Athens, Greece); D. Maharjan (Kathmandu Medical College, Nepal); D. Malde (Leicester Hospitals, Leicester, UK); M. Mankarious (Penn State College of Medicine, Pennsylvania, USA); G. Martel (University of Ottawa, Ottawa, Canada); J. Martin (University of South Carolina, South Carolina, USA); M. Mazzola (ASST Grande Ospedale Metropolitano Niguarda, Italy); A. Mehrabi (Ruprecht-Karls University, Heidelberg, Germany); R. Memeo (Regional General Hospital F. Miulli, Bari, Italy); F. Milana (Humanitas Hospital, Milan, Italy); G. Molina (Brigham and Women’s Hospital, Boston, USA); L. Monette (University of Ottawa, Ottawa, Canada); H. Morgul (University of Münster, Münster, Germany); D. Moris (Laiko General Hospital and Kapodistrian University of Athens, Greece); A. Morsi-Yeroyannis (Ippokratio General Hospital and University Clinic Thessaloiniki, Thessaloniki, Greece); N. Mowbray (University Hospital of Wales, Cardiff, UK); F. Mulita (University of Patras, Rion, Greece); E. Maria Muttillo (Sapienza University of Rome, Italy); M. Nandasena (Colombo South Teaching Hospital, Colombo, Sri Lanka); P. Rashid Nashidengo (Windhoek Central Hospital, Windhoek, Namibia); A. Nickkholgh (Ruprecht-Karls University, Heidelberg, Germany); C. Byron Noel (Universitas Academic Hospital, Bloemfontein, South Africa); M. Ohtsuka (Chiba University, Chiba, Japan); A. Ozolins (Pauls Stradina Clinical University Hospital, Riga, Latvia); S. Pandanaboyana (Newcastle University Hospitals, Newcastle, UK); N. Pararas (Dr Sulaiman Al Habib Hospital, Riyadh, Saudi Arabia); A. Parente (University Hospitals Birmingham, Birmingham, UK); J. Peng (Penn State College of Medicine, Pennsylvania, USA); A. Perfecto Valero (Cruces University Hospital, Barakaldo, Spain); J. Perinel (Edouard Herriot Hospital, Lyon, France); K. Perivoliotis (University Hospital of Larissa, Larissa, Greece); T. Perra (AOU Sassari, Sardinia, Italy); P. Pessaux (Nouvel Hospital Civil, Strasbourg, France); N. Petruch (University Medical Center, Lübeck, Germany); G. Piccolo (San Paolo Hospital, University of Milan, Milan, Italy); L. Piros (Semmelweis University, Budapest, Hungary); A. Porcu (AOU Sassari, Sardinia, Italy); V. Prabakaran (Newcastle University Hospitals, Newcastle, UK); R. Prasad (Leeds Teaching Hospitals NHS Trust, Leeds, UK); M. Prieto Calvo (Cruces University Hospital, Barakaldo, Spain); F. Primavesi (Salzkammergut Klinikum, Vöcklabruck, Austria); E. Maria Pueyo Periz (Hospital Virgen Del Rocio, Spain); A. Quaglia (Royal Free Hospital, London, UK); J. Ramia Angel (General Universitario de Alicante, Alicante, Spain); A. Rammohan (Rela Hospital, Chennai, India); F. Razionale (Fondazione Policlinico Universitario A. Gemelli, Rome, Italy); R. Robles Campos (Clinic and University Virgen de la Arrixaca Hospital, Murcia, Spain); M. Roy (Tata Medical Centre, Kolkata, India); S. Rozwadowski (University Hospital Bristol, Bristol, UK); L. Ruffolo (University of Rochester Medical College, New York, USA); N. Ruiz (Hospital Clínico Regional Dr Guillermo Grant Benavente, Concepcion, Chile); A. Ruzzenante (University of Verona Medical School, Verona, Italy); L. Saadat (Brigham and Women’s Hospital, Boston, USA); M. Amine Said (Sahloul Hospital, Tunisia); E. Saladino (AO Papardo, Messina, Italy); G. Saliba (Karolinska University Hospital, Stockholm, Sweden); P. Sandstrom (University Hospital Linkoping, Sweden); C. Alberto Schena (Regional General Hospital F. Miulli, Bari, Italy); A. Scholer (University of South Carolina, South Carolina, USA); C. Schwarz (University of Medicine Vienna, Austria); L. Serafini (University of Bologna, Bologna, Italy); L. Serrablo (Hospital Miguel Servet, Zaragoza, Spain); P. Serrano (McMaster University, Hamilton, Ontario, Canada); D. Sharma (Tribhuvan University Teaching Hospital, Kathmandu, Nepal); A. Sheen (Manchester Foundation NHS Trust, Manchester, UK); V. Siddagangaiah (Royal Blackburn Hospital, Blackburn, UK); M. Silva (Oxford University Hospitals NHS Foundation Trust, Oxford, UK); S. Singh (King George’s Medical University, Lucknow, India); A. Siriwardena (Manchester Foundation NHS Trust, Manchester, UK); M. Skalski (Medical University of Warsaw, Warsaw, Poland); M. Smig (Vilnius University Hospital, Vilnius, Lithuania); F. Soliman (Morriston Hospital, Swansea, UK); A. Arun Sonkar (King George’s Medical University, Lucknow, India); D. Sousa Silva (Centro Hospitalar Universitário do Porto, Porto, Portugal); E. Sparrelid (Karolinska University Hospital, Stockholm, Sweden); H. Spiers (Cambridge University Hospitals, Cambridge, UK); P. Srinivasan (Kings College London, London, UK); M. Sternby Eilard (Sahlgrenska University Hospital, Gothenburg, Sweden); O. Strobel (University of Medicine Vienna, Austria); U. Stupan (University Medical Centre Ljubljana, Ljubljana, Slovenia); M. Angel Suarez-Munoz (University Hospital Virgen de la Victoria, Malaga, Spain); M. Subramaniam (Hospital Sultanah Bahiyah, Alor Setar, Malaysia); T. Sugiura (Shizuoka Cancer Centre, Shizuoka, Japan); R. Sutcliffe (University Hospitals Birmingham, Birmingham, UK); H. Swank (OLVG, Amsterdam, Netherlands); S. Talukder (Cambridge University Hospitals, Cambridge, UK); L. Taylor (Melbourne Surgical Group, Melbourne, Australia); P. Bikram Thapa (Kathmandu Medical College, Nepal); C. Teh (National Kidney and Transplant Institute, Manila, Philippines); A. Thepbunchonchai (Rajavithi Hospital, Bangkok, Thailand); C. Thieu (McMaster University, Hamilton, Ontario, Canada); N. Tiwari (Newcastle University Hospitals, Newcastle, UK); G. Torzilli (Humanitas Hospital, Milan, Italy); C. Tovikkai (Mahidol University, Bangkok, Thailand); B. Trotovsek (University Medical Centre Ljubljana, Ljubljana, Slovenia); S. Tsaramanidis (Ippokratio General Hospital and University Clinic Thessaloiniki, Thessaloniki, Greece); G. Tsoulfas (Ippokratio General Hospital and University Clinic Thessaloiniki, Thessaloniki, Greece); K. Uesaka (Shizuoka Cancer Centre, Shizuoka, Japan); G. Umar (Aminu Kano Teaching Hospital, Kano, Nigeria); L. Urbani (Azienda Ospedaliero Universitaria Pisana, Pisa, Italy); M. Vailas (University of Patras, Rion, Greece); R. van Dam (MUMC Maastricht, Maastricht, Netherlands); P. van de Boezem (Radboud UMC, Nijmegen, Netherlands); S. van Laarhoven (University Hospital Bristol, Bristol, UK); T. Vanagas (Hospital of Lithuanian University of Health Sciences Kauno Klinikos, Kaunas, Lithuania); M. Van Dooren (Radboud UMC, Nijmegen, Netherlands); M. Viennet (Dijon University Hospital, Dijon, France); L. Vigano (Humanitas Hospital, Milan, Italy); A. Vijayashanker (Kings College London, London, UK); C. Villodre (General Universitario de Alicante, Alicante, Spain); T. Wakai (Niigata University, Niigata, Japan); A. Workneh (University of Ottawa, Ottawa, Canada); L. Xu (China Japan Friendship Hospital, Beijing, China); M. Yamamoto (Tokyo Women’s Medical Hospital, Tokyo, Japan); Z. Yang (China Japan Friendship Hospital, Beijing, China); R. Young (Royal Liverpool University Hospital, Liverpool, UK); M. Zivanovic (University Clinical Center of Serbia, Belgrade, Serbia).

## Supplementary Material

zrag052_Supplementary_Data

## Data Availability

Data ownership for the data compiled in the OMEGA study remains the property of individual OMEGA collaborating units, and data sharing with third parties will therefore not be possible without permission from respective individual contributors.

## Supplementary material

*Supplementary materia*l is available at *BJS Open* online.
