# Supplementary material for: Perioperative outcomes and long-term survival following resection of gallbladder cancer in older adults: retrospective study
Source: BJS Open. 2026 Jul 31;10(4):zrag052. doi: 10.1093/bjsopen/zrag052 (PMC13426238; doi:10.1093/bjsopen/zrag052)
Supplement: zrag052_Supplementary_Data [file zrag052_supplementary_data.docx]

**Perioperative outcomes and long-term survival following resection of gallbladder cancer in elderly patients**

Anita Balakrishnan PhD FRCS^a^, Petros Barmpounakis PhD^b^, Nikolaos Demiris PhD^b^, Bodil Andersson MD PhD^c^, Alejandro Brañes MD^d^, Xavier de Aretxabala MD^e^, Paul Gibbs MD FRCS^a^, Simon J F Harper MD FRCS^a^, Emmanuel L Huguet PhD FRCS^a^, Asif Jah FRCS^a^, Vasilis Kosmoliaptsis PhD FRCS^a^, Javier Lendoire MD PhD^f^, Siong S Liau MD FRCS^a^, Shishir K Maithel MD^g^, Jack L Martin PhD FRCS^a^, Colin Noel MD, MMed^h^, Raaj K Praseedom FRCS^a^, Alejandro Serrablo MD PhD^i^, Volkan Adsay MD^j^ for the OMEGA study investigators

^a^Department of HPB Surgery, Cambridge University Hospitals NHS Foundation Trust, and Department of Surgery, University of Cambridge, Hills Road, Cambridge, CB2 0QQ, United Kingdom

^b^Cambridge Clinical Trials Unit – Cancer Theme, Cambridge University Hospitals NHS Foundation Trust, Hills Road, Cambridge, CB2 0QQ, United Kingdom, and Department of Statistics, Athens University of Economics and Business, Athens, Greece

^c^Department of Surgery, Lund University, Skane University Hospital, Lund, Sweden

^d^Department of HPB Surgery, Hospital Dr Sotero del Rio, Santiago, Chile

^e^Department of Digestive Surgery, Hepato-Pancreato-Biliary Surgery Unit, Surgery Service, Gallbladder Consortium Chile, Sotero del Rio Hospital and Clinica Alemana, Santiago, Chile

^f^Department of Surgery, University of Buenos Aires, Hospital Dr Cosme Argerich, Buenos Aires, Argentina

^g^Division of Surgical Oncology, Robert H Lurie Comprehensive Cancer Centre, Northwestern University, Illinois, United States of America

^h^Gastrointestinal Surgery and HPB Surgery, Department of Surgery, University of the Free State, Bloemfontein, South Africa

^i^Department of HPB Surgery, Miguel Servet University Hospital, Zaragoza, Spain

^j^Department of Pathology, Koç University Hospital, Istanbul 34010, Turkey; Koç University Research Center for Translational Medicine (KUTTAM), Istanbul 34010, Turkey

Corresponding author:

Anita Balakrishnan, PhD FRCS

Consultant Hepatopancreatobiliary Surgeon, Department of HPB Surgery, Cambridge University Hospitals NHS Foundation Trust, Hills Road, Cambridge, CB2 0QQ, United Kingdom

Email: ab2031@cam.ac.uk

Tel: +44 1223 256 151

**Supplementary Materials - Index**

| **Supplementary Methods** | |  |  |
| --- | --- | --- | --- |
| Statistical methods | | *pag. 4* |  |
| **Supplementary Figures** | |  |  |
| Supplementary Figure 1: Distribution of countries participating in the OMEGA study (marked by red squares) superimposed on estimated global age-standardized incidence rates (ASR) of gallbladder cancer per 100,000 individuals. | | *pag. 5* |  |
| Supplementary Table 1: Missingness table for all analysed variables by age cohort Supplementary Figure 2: a) Cox regression for OS on the unmatched dataset with age interaction with income level and b) Cox regression for RFS on the unmatched dataset with age interaction with income level Supplementary Figure 3:a) 90-day landmark analysis and b) 120-day landmark analysis Cox regression on the unmatched and matched datasets on OS  Supplementary Figure 4: Cox regression analysis for a) OS and b) RFS, excluding all patients with Nx status  Supplementary Figure 5: Cox regression analysis for OS using continuous age with splines  Supplementary Figure 6: Cox regression analysis of the matched and unmatched cohorts for OS with cut-off of a)>70 years and b)>80 years  Supplementary Figure 7: a) 90-day landmark analysis and b) 120-day landmark analysis Cox regression on the unmatched and matched datasets on RFS | | *pag. 6*  *pag 8*  *pag 10*  *pag 12*  *pag 14*  *pag 15*  *pag 17* |  |
| Supplementary Figure 8: Cox regression analysis for RFS using continuous age with splines  Supplementary Figure 9: Cox regression analysis of the matched and unmatched cohorts for RFS with cut-off of a)>70 years and b)>80 years  Supplementary Figure 10: Multivariable logistic regression of factors associated with 1-year OS in the full cohort | | *pag. 19*  *pag 20*  *pag 22* |  |
| Supplementary Figure 11: Multivariable logistic regression of factors associated with 1-year OS in the >75 year cohort  Supplementary Figure 12:a) 90-day landmark analysis and b) 120-day landmark analysis Cox regression on OS in the in the >75 year cohort | | *pag. 23*  *pag 24* | |
| Supplementary Figure 13: Multivariable logistic regression of factors associated with complications in the >75 year cohort  Supplementary Table 2: Causes of 90-day mortality in older and younger adults | | *pag. 26*  *pag 27* |  |
|  | |  |  |

**Supplementary Methods**

**Statistical Methods:**

Matching was implemented using the MatchIt package in R, and balance diagnostics were assessed using standardized mean differences (SMDs) across covariates, with an SMD < 0.1 considered indicative of adequate balance. The 'treated' group was considered the older group (people >75 years old), and average treatment effects on treated (ATT) were estimated. The optimal and nearest-neighbour matching techniques produced one matched pair for every patient in the treated group without replacement. The full matching algorithm matched multiple persons from the younger group to each person in the older group, also without replacement.  Matching weights derived from the respective algorithms were incorporated in all subsequent outcome models to account for variable matching ratios.

All analyses were conducted using the matched cohorts and corresponding weights to obtain unbiased estimates of treatment effects under the assumption of not having any unobserved confounders. Time-to-event outcomes, including OS and RFS, were analysed using weighted Cox proportional hazards models, using as explanatory variable the age group and incorporating the matching weights. For binary outcomes, including 1-year survival and the incidence of post-operative complications, weighted logistic regression models were used. The incidence of post-operative complications model was only analysed using baseline pre-operative characteristics (gender, extent of surgery, income level and comorbidities) as late post-operative variables such as chemotherapy and radiotherapy would not be expected to affect post-surgical complications. Post-operative variables were also omitted for a sensitivity analysis of OS, RFS and 1-year OS. 90-day and 120-day landmark analyses of OS and RFS for the whole cohort were additionally performed as sensitivity analysis to mitigate against any early non-cancer mortality bias. Age was also analysed as a continuous variable with restricted cubic splines, and using additional cut-offs of >70 years and >80 years to identify any non-linear association between age and risk. The regression analyses was repeated for OS and RFS excluding patients with Nx stage to mitigate any influence of inadequately staged patients on the findings. 90-day and 120-day landmark analyses was also performed for OS and RFS for the older adult cohort separately to assess for confounding by indication. Interaction testing was performed between age and income level to explore any associations or heterogeneity.

**Supplementary Figures**


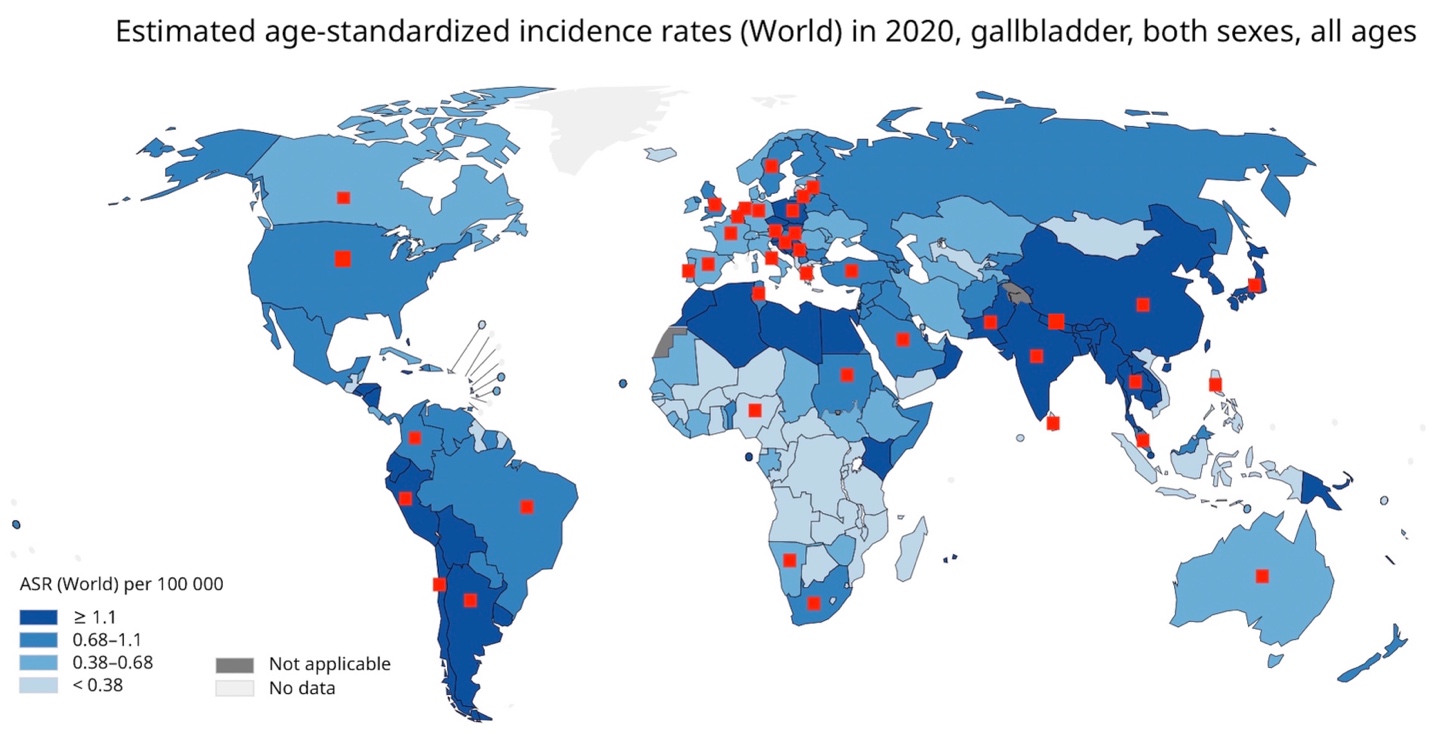


Supplementary Figure 1: Distribution of countries participating in the OMEGA study (marked by red squares) superimposed on estimated global age standardized incidence rates (ASR) of gallbladder cancer per 100,000 individuals. Adapted with permission from the International Agency for Research on Cancer’s GLOBOCAN database (reference 1).

| Variable | <75 years | >75 years | Total |
| --- | --- | --- | --- |
| Sex | 0 (0%) | 0 (0%) | 0 (0%) |
| Age | 0 (0%) | 0 (0%) | 3 (0.082%) |
| R status | 10 (0.359%) | 7 (0.791%) | 17 (0.462%) |
| Adjuvant Chemotherapy | 169 (6.062%) | 42 (4.746%) | 211 (5.74%) |
| Adjuvant Radiotherapy | 166 (5.954%) | 41 (4.633%) | 207 (5.631%) |
| Income Level | 0 (0%) | 0 (0%) | 0 (0%) |
| T Stage | 0 (0%) | 0 (0%) | 0 (0%) |
| N Stage | 0 (0%) | 0 (0%) | 0 (0%) |
| Extent of Surgery | 0 (0%) | 0 (0%) | 2 (0.054%) |
| Complication | 38 (1.363%) | 21 (2.373%) | 61 (1.659%) |
| Os Time | 44 (1.578%) | 6 (0.678%) | 52 (1.415%) |
| Os Event | 44 (1.578%) | 6 (0.678%) | 52 (1.415%) |
| Rfs Time | 489 (17.539%) | 193 (21.808%) | 685 (18.634%) |
| Rfs Event | 488 (17.504%) | 193 (21.808%) | 684 (18.607%) |
| CCI Score | 181 (6.492%) | 40 (4.52%) | 222 (6.039%) |

Supplementary Table 1: Missingness table for all analysed variables by age cohort

| **Variable** | **HR** | **95% CI** | **p-value** |
| --- | --- | --- | --- |
| **Age Bracket** |  |  |  |
| <75 | — | — |  |
| >=75 | 2.30 | 1.39, 3.79 | 0.001 |
| **Income Level** |  |  |  |
| NHI | — | — |  |
| HI | 1.27 | 1.07, 1.52 | 0.008 |
| **sex** |  |  |  |
| Female | — | — |  |
| Male | 0.93 | 0.82, 1.06 | 0.3 |
| **R status** |  |  |  |
| R0 | — | — |  |
| R1 | 1.63 | 1.38, 1.93 | <0.001 |
| **Adjuvant Chemo** |  |  |  |
| No | — | — |  |
| Yes | 0.89 | 0.77, 1.03 | 0.12 |
| **Adjuvant radiotherapy** |  |  |  |
| No | — | — |  |
| Yes | 0.93 | 0.72, 1.20 | 0.6 |
| **T Stage** |  |  |  |
| T1a | — | — |  |
| T1b | 1.76 | 1.0, 3.12 | 0.052 |
| T2 | 3.92 | 2.34, 6.59 | <0.001 |
| T3 | 10.7 | 6.31, 18.3 | <0.001 |
| T4 | 10.6 | 5.97, 19.0 | <0.001 |
| **N Stage** |  |  |  |
| N0 | — | — |  |
| N1 | 1.86 | 1.59, 2.17 | <0.001 |
| N2 | 3.32 | 2.66, 4.14 | <0.001 |
| Nx | 1.92 | 1.51, 2.43 | <0.001 |
| **Extent of Surgery** |  |  |  |
| CO | — | — |  |
| LLR | 0.89 | 0.69, 1.15 | 0.4 |
| MR | 0.97 | 0.74, 1.27 | 0.8 |
| **CCI Bracket** |  |  |  |
| <5 | — | — |  |
| >5 | 1.09 | 0.95, 1.25 | 0.2 |
| **Age Bracket * income Level** |  |  |  |
| >=75 * HI | 0.56 | 0.33, 0.93 | 0.026 |
| Abbreviations: CI = Confidence Interval, HR = Hazard Ratio | | | |

### Supplementary Figure 2: a) Cox regression for OS on the unmatched dataset with age interaction with income level

| **Variable** | **HR** | **95% CI** | **p-value** |
| --- | --- | --- | --- |
| **Age Bracket** |  |  |  |
| <75 | — | — |  |
| >=75 | 2.17 | 1.28, 3.70 | 0.004 |
| **Income Level** |  |  |  |
| Non-high income | — | — |  |
| High income | 1.26 | 1.08, 1.48 | 0.004 |
| **Sex** |  |  |  |
| Female | — | — |  |
| Male | 0.90 | 0.80, 1.02 | 0.10 |
| **R status** |  |  |  |
| R0 | — | — |  |
| R1 | 1.86 | 1.58, 2.18 | <0.001 |
| **Adjuvant Chemo** |  |  |  |
| No | — | — |  |
| Yes | 0.99 | 0.86, 1.13 | 0.9 |
| **Adjuvant radiotherapy** |  |  |  |
| No | — | — |  |
| Yes | 1.07 | 0.87, 1.32 | 0.5 |
| **T Stage** |  |  |  |
| T1a | — | — |  |
| T1b | 3.28 | 1.55, 6.92 | 0.002 |
| T2 | 7.71 | 3.80, 15.6 | <0.001 |
| T3 | 18.4 | 9.00, 37.6 | <0.001 |
| T4 | 16.8 | 7.97, 35.5 | <0.001 |
| **N Stage** |  |  |  |
| N0 | — | — |  |
| N1 | 1.88 | 1.63, 2.17 | <0.001 |
| N2 | 3.07 | 2.51, 3.75 | <0.001 |
| Nx | 1.94 | 1.56, 2.42 | <0.001 |
| **Extent of Surgery** |  |  |  |
| CO | — | — |  |
| LLR | 1.08 | 0.84, 1.38 | 0.6 |
| MR | 1.16 | 0.89, 1.50 | 0.3 |
| **CCI Bracket** |  |  |  |
| <5 | — | — |  |
| >5 | 1.22 | 1.07, 1.38 | 0.002 |
| **Age Bracket * income Level** |  |  |  |
| >=75 * HI | 0.46 | 0.27, 0.80 | 0.006 |
| Abbreviations: CI = Confidence Interval, HR = Hazard Ratio | | | |

### Supplementary Figure 2: b) Cox regression for RFS on the unmatched dataset with age interaction with income level

| **Variable** | **HR** | **95% CI** | **p-value** |
| --- | --- | --- | --- |
| **Age Bracket** |  |  |  |
| <75 | — | — |  |
| >=75 | 1.37 | 1.16, 1.62 | <0.001 |
| **sex** |  |  |  |
| Female | — | — |  |
| Male | 0.94 | 0.82, 1.08 | 0.4 |
| **R status** |  |  |  |
| R0 | — | — |  |
| R1 | 1.69 | 1.41, 2.03 | <0.001 |
| **Adjuvant Chemo** |  |  |  |
| No | — | — |  |
| Yes | 1.04 | 0.89, 1.22 | 0.6 |
| **Adjuvant radiotherapy** |  |  |  |
| No | — | — |  |
| Yes | 0.98 | 0.75, 1.28 | 0.9 |
| **Income Level** |  |  |  |
| NHI | — | — |  |
| HI | 1.18 | 0.99, 1.41 | 0.067 |
| **T Stage** |  |  |  |
| T1a | — | — |  |
| T1b | 2.30 | 1.22, 4.36 | 0.010 |
| T2 | 4.95 | 2.75, 8.91 | <0.001 |
| T3 | 12.1 | 6.63, 22.1 | <0.001 |
| T4 | 11.8 | 6.17, 22.7 | <0.001 |
| **N Stage** |  |  |  |
| N0 | — | — |  |
| N1 | 1.83 | 1.55, 2.15 | <0.001 |
| N2 | 3.30 | 2.58, 4.21 | <0.001 |
| Nx | 1.94 | 1.51, 2.49 | <0.001 |
| **Extent of Surgery** |  |  |  |
| CO | — | — |  |
| LLR | 0.87 | 0.67, 1.14 | 0.3 |
| MR | 0.95 | 0.72, 1.27 | 0.7 |
| **CCI Bracket** |  |  |  |
| <5 | — | — |  |
| >5 | 1.10 | 0.95, 1.28 | 0.2 |
| Abbreviations: CI = Confidence Interval, HR = Hazard Ratio | | | |

|  | **Optimal** | | | **Nearest** | | |
| --- | --- | --- | --- | --- | --- | --- |
| **Variable** | **HR** | **95% CI** | **p-value** | **HR** | **95% CI** | **p-value** |
| **age_boolean** | 1.37 | 1.15, 1.64 | <0.001 | 1.34 | 1.12, 1.60 | 0.001 |

Supplementary Figure 3:a) 90-day landmark analysis on the unmatched and matched datasets on OS

| **Variable** | **HR** | **95% CI** | **p-value** |
| --- | --- | --- | --- |
| **ageBracket** |  |  |  |
| <75 | — | — |  |
| >=75 | 1.37 | 1.15, 1.63 | <0.001 |
| **sex** |  |  |  |
| Female | — | — |  |
| Male | 0.96 | 0.83, 1.10 | 0.5 |
| **R status** |  |  |  |
| R0 | — | — |  |
| R1 | 1.64 | 1.35, 1.98 | <0.001 |
| **Adjuvant Chemo** |  |  |  |
| No | — | — |  |
| Yes | 1.08 | 0.92, 1.26 | 0.4 |
| **Adjuvant radiotherapy** |  |  |  |
| No | — | — |  |
| Yes | 0.98 | 0.75, 1.30 | >0.9 |
| **Income Level** |  |  |  |
| NHI | — | — |  |
| HI | 1.22 | 1.01, 1.48 | 0.037 |
| **T Stage** |  |  |  |
| T1a | — | — |  |
| T1b | 2.28 | 1.20, 4.33 | 0.012 |
| T2 | 4.89 | 2.70, 8.84 | <0.001 |
| T3 | 11.9 | 6.48, 21.9 | <0.001 |
| T4 | 11.6 | 5.98, 22.4 | <0.001 |
| **N Stage** |  |  |  |
| N0 | — | — |  |
| N1 | 1.82 | 1.54, 2.16 | <0.001 |
| N2 | 3.44 | 2.66, 4.45 | <0.001 |
| Nx | 1.94 | 1.50, 2.51 | <0.001 |
| **Extent of Surgery** |  |  |  |
| CO | — | — |  |
| LLR | 0.85 | 0.65, 1.12 | 0.3 |
| MR | 0.88 | 0.66, 1.18 | 0.4 |
| **CCI Bracket** |  |  |  |
| <5 | — | — |  |
| >5 | 1.12 | 0.96, 1.30 | 0.2 |
| Abbreviations: CI = Confidence Interval, HR = Hazard Ratio | | | |

|  | **Optimal** | | | **Nearest** | | |
| --- | --- | --- | --- | --- | --- | --- |
| **Variable** | **HR** | **95% CI** | **p-value** | **HR** | **95% CI** | **p-value** |
| **age_boolean** | 1.36 | 1.13, 1.63 | <0.001 | 1.42 | 1.18, 1.71 | <0.001 |

Supplementary Figure 3:b) 120-day landmark analysis on the unmatched and matched datasets on OS

| **Variable** | **HR** | **95% CI** | **p-value** |
| --- | --- | --- | --- |
| **ageBracket** |  |  |  |
| <75 | — | — |  |
| >75 | 1.29 | 1.09, 1.53 | 0.003 |
| **sex** |  |  |  |
| Female | — | — |  |
| Male | 0.91 | 0.79, 1.05 | 0.2 |
| **R status** |  |  |  |
| R0 | — | — |  |
| R1 | 1.55 | 1.29, 1.86 | <0.001 |
| **Adjuvant Chemo** |  |  |  |
| No | — | — |  |
| Yes | 0.89 | 0.76, 1.03 | 0.13 |
| **Adjuvant radiotherapy** |  |  |  |
| No | — | — |  |
| Yes | 0.91 | 0.69, 1.19 | 0.5 |
| **Income Level** |  |  |  |
| NHI | — | — |  |
| HI | 1.21 | 1.01, 1.45 | 0.034 |
| **T Stage** |  |  |  |
| T1a | — | — |  |
| T1b | 2.08 | 0.92, 4.69 | 0.079 |
| T2 | 3.85 | 1.77, 8.36 | <0.001 |
| T3 | 10.4 | 4.75, 23.0 | <0.001 |
| T4 | 9.99 | 4.40, 22.7 | <0.001 |
| **N Stage** |  |  |  |
| N0 | — | — |  |
| N1 | 1.91 | 1.64, 2.24 | <0.001 |
| N2 | 3.44 | 2.75, 4.30 | <0.001 |
| **Extent of Surgery** |  |  |  |
| CO | — | — |  |
| LLR | 0.81 | 0.59, 1.10 | 0.2 |
| MR | 0.89 | 0.64, 1.23 | 0.5 |
| **CCI Bracket** |  |  |  |
| <5 | — | — |  |
| >5 | 1.10 | 0.95, 1.27 | 0.2 |
| Abbreviations: CI = Confidence Interval, HR = Hazard Ratio | | | |

|  | **Optimal** | | | **Nearest** | | |
| --- | --- | --- | --- | --- | --- | --- |
| **Variable** | **HR** | **95% CI** | **p-value** | **HR** | **95% CI** | **p-value** |
| **age_boolean** | 1.28 | 1.07, 1.53 | 0.008 | 1.22 | 1.03, 1.45 | 0.024 |

Supplemental Figure 4:a) Cox regression analysis for OS excluding all patients with Nx status

| **Variable** | **HR** | **95% CI** | **p-value** |
| --- | --- | --- | --- |
| **Age Bracket** |  |  |  |
| <75 | — | — |  |
| >75 | 1.06 | 0.90, 1.25 | 0.5 |
| **sex** |  |  |  |
| Female | — | — |  |
| Male | 0.90 | 0.79, 1.02 | 0.093 |
| **R status** |  |  |  |
| R0 | — | — |  |
| R1 | 1.79 | 1.50, 2.13 | <0.001 |
| **Adjuvant Chemo** |  |  |  |
| No | — | — |  |
| Yes | 0.97 | 0.84, 1.12 | 0.7 |
| **Adjuvant radiotherapy** |  |  |  |
| No | — | — |  |
| Yes | 1.02 | 0.82, 1.27 | 0.8 |
| **Income Level** |  |  |  |
| NHI | — | — |  |
| HI | 1.19 | 1.01, 1.40 | 0.036 |
| **T Stage** |  |  |  |
| T1a | — | — |  |
| T1b | 3.82 | 1.19, 12.3 | 0.025 |
| T2 | 8.73 | 2.80, 27.2 | <0.001 |
| T3 | 20.8 | 6.63, 65.3 | <0.001 |
| T4 | 18.5 | 5.76, 59.3 | <0.001 |
| **N Stage** |  |  |  |
| N0 | — | — |  |
| N1 | 1.89 | 1.64, 2.18 | <0.001 |
| N2 | 3.06 | 2.50, 3.75 | <0.001 |
| **Extent of Surgery** |  |  |  |
| CO | — | — |  |
| LLR | 1.06 | 0.75, 1.49 | 0.8 |
| MR | 1.17 | 0.82, 1.66 | 0.4 |
| **CCI Bracket** |  |  |  |
| <5 | — | — |  |
| >5 | 1.21 | 1.06, 1.39 | 0.004 |
| Abbreviations: CI = Confidence Interval, HR = Hazard Ratio | | | |

|  | **Optimal** | | | **Nearest** | | |
| --- | --- | --- | --- | --- | --- | --- |
| **Variable** | **HR** | **95% CI** | **p-value** | **HR** | **95% CI** | **p-value** |
| **age_boolean** | 1.05 | 0.89, 1.24 | 0.6 | 1.00 | 0.85, 1.17 | >0.9 |

Supplemental Figure 4:b) Cox regression analysis for RFS excluding all patients with Nx status

| **term** | **HR** | **95% CI** | **p-value** |
| --- | --- | --- | --- |
| pspline(Age, df = 2), lin | 1.01 | - | <0.001 |
| pspline(Age, df = 2), non | - | - | 0.007 |
| Sex Male | 0.92 | 0.81, 1.05 | 0.228 |
| R1 | 1.66 | 1.40, 1.95 | <0.001 |
| Adjuvant Chemo Yes | 0.91 | 0.79, 1.05 | 0.196 |
| Adjuvant RT Yes | 0.92 | 0.71, 1.20 | 0.532 |
| Income Level HI | 1.15 | 0.96, 1.37 | 0.125 |
| T1b | 1.75 | 0.99, 3.11 | 0.055 |
| T2 | 3.83 | 2.28, 6.44 | <0.001 |
| T3 | 10.43 | 6.12, 17.79 | <0.001 |
| T4 | 10.43 | 5.86, 18.59 | <0.001 |
| N1 | 1.85 | 1.59, 2.16 | <0.001 |
| N2 | 3.34 | 2.68, 4.17 | <0.001 |
| Nx | 1.94 | 1.54, 2.45 | <0.001 |
| Extent Surgery LLR | 0.90 | 0.70, 1.15 | 0.408 |
| Extent Surgery MR | 0.98 | 0.75, 1.28 | 0.897 |
| CCI Bracket>5 | 1.04 | 0.90, 1.21 | 0.577 |


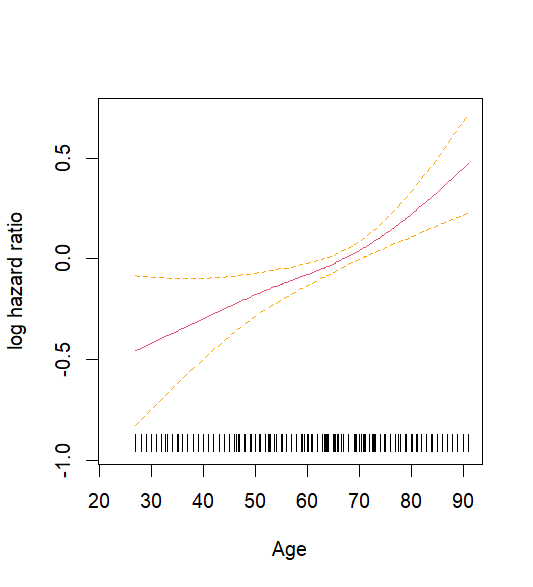


Supplementary Figure 5: Cox regression analysis for OS using continuous age with splines

| **Variable** | **HR** | **95% CI** | **p-value** |
| --- | --- | --- | --- |
| **age** |  |  |  |
| <70 | — | — |  |
| >70 | 1.18 | 1.01, 1.37 | 0.035 |
| **sex** |  |  |  |
| Female | — | — |  |
| Male | 0.92 | 0.81, 1.05 | 0.2 |
| **Rstatus** |  |  |  |
| R0 | — | — |  |
| R1 | 1.64 | 1.38, 1.93 | <0.001 |
| **Adjuvant Chemo** |  |  |  |
| No | — | — |  |
| Yes | 0.89 | 0.77, 1.02 | 0.10 |
| **Adjuvant radiotherapy** |  |  |  |
| No | — | — |  |
| Yes | 0.93 | 0.72, 1.20 | 0.6 |
| **Income Level** |  |  |  |
| NHI | — | — |  |
| HI | 1.22 | 1.02, 1.44 | 0.026 |
| **T Stage** |  |  |  |
| T1a | — | — |  |
| T1b | 1.79 | 1.01, 3.18 | 0.048 |
| T2 | 3.99 | 2.37, 6.73 | <0.001 |
| T3 | 10.9 | 6.37, 18.6 | <0.001 |
| T4 | 10.7 | 5.99, 19.1 | <0.001 |
| **N Stage** |  |  |  |
| N0 | — | — |  |
| N1 | 1.85 | 1.59, 2.16 | <0.001 |
| N2 | 3.32 | 2.66, 4.14 | <0.001 |
| Nx | 1.97 | 1.56, 2.48 | <0.001 |
| **Extent of Surgery** |  |  |  |
| CO | — | — |  |
| LLR | 0.87 | 0.68, 1.12 | 0.3 |
| MR | 0.95 | 0.73, 1.23 | 0.7 |
| **CCI Bracket** |  |  |  |
| <5 | — | — |  |
| >5 | 1.10 | 0.94, 1.27 | 0.2 |
| Abbreviations: CI = Confidence Interval, HR = Hazard Ratio | | | |

|  | **Optimal** | | | **Nearest** | | |
| --- | --- | --- | --- | --- | --- | --- |
| **Variable** | **HR** | **95% CI** | **p-value** | **HR** | **95% CI** | **p-value** |
| **age_boolean** | 1.19 | 1.04, 1.35 | 0.009 | 1.19 | 1.04, 1.35 | 0.009 |

Supplementary Figure 6a: Cox regression analysis of the matched and unmatched cohorts for OS with cut-off of >70 years

| **Variable** | **HR** | **95% CI** | **p-value** |
| --- | --- | --- | --- |
| **age** |  |  |  |
| <80 | — | — |  |
| >80 | 1.51 | 1.24, 1.84 | <0.001 |
| **sex** |  |  |  |
| Female | — | — |  |
| Male | 0.93 | 0.82, 1.06 | 0.3 |
| **R status** |  |  |  |
| R0 | — | — |  |
| R1 | 1.62 | 1.38, 1.92 | <0.001 |
| **Adjuvant Chemo** |  |  |  |
| No | — | — |  |
| Yes | 0.90 | 0.78, 1.04 | 0.2 |
| **Adjuvant radiotherapy** |  |  |  |
| No | — | — |  |
| Yes | 0.92 | 0.71, 1.19 | 0.5 |
| **Income Level** |  |  |  |
| NHI | — | — |  |
| HI | 1.23 | 1.04, 1.46 | 0.016 |
| **Tumour Stage** |  |  |  |
| T1a | — | — |  |
| T1b | 1.77 | 0.99, 3.14 | 0.053 |
| T2 | 3.93 | 2.34, 6.60 | <0.001 |
| T3 | 10.7 | 6.30, 18.3 | <0.001 |
| T4 | 10.4 | 5.86, 18.6 | <0.001 |
| **N Stage** |  |  |  |
| N0 | — | — |  |
| N1 | 1.86 | 1.59, 2.16 | <0.001 |
| N2 | 3.35 | 2.69, 4.17 | <0.001 |
| Nx | 1.94 | 1.54, 2.44 | <0.001 |
| **Extent of Surgery** |  |  |  |
| CO | — | — |  |
| LLR | 0.91 | 0.71, 1.17 | 0.5 |
| MR | 1.00 | 0.77, 1.31 | >0.9 |
| **CCI Bracket** |  |  |  |
| <5 | — | — |  |
| >5 | 1.13 | 0.99, 1.29 | 0.073 |
| Abbreviations: CI = Confidence Interval, HR = Hazard Ratio | | | |

|  | **Optimal** | | | **Nearest** | | |
| --- | --- | --- | --- | --- | --- | --- |
| **Variable** | **HR** | **95% CI** | **p-value** | **HR** | **95% CI** | **p-value** |
| **age_boolean** | 1.44 | 1.12, 1.85 | 0.005 | 1.50 | 1.19, 1.89 | <0.001 |

Supplementary Figure 6b: Cox regression analysis of the matched and unmatched cohorts for OS with cut-off of >80 years

| **Variable** | **HR** | **95% CI** | **p-value** |
| --- | --- | --- | --- |
| **Age Bracket** |  |  |  |
| <75 | — | — |  |
| >=75 | 1.11 | 0.95, 1.30 | 0.2 |
| **sex** |  |  |  |
| Female | — | — |  |
| Male | 0.90 | 0.80, 1.03 | 0.13 |
| **R status** |  |  |  |
| R0 | — | — |  |
| R1 | 1.93 | 1.63, 2.28 | <0.001 |
| **Adjuvant Chemo** |  |  |  |
| No | — | — |  |
| Yes | 1.17 | 1.01, 1.34 | 0.032 |
| **Adjuvant radiotherapy** |  |  |  |
| No | — | — |  |
| Yes | 1.13 | 0.91, 1.39 | 0.3 |
| **Income Level** |  |  |  |
| NHI | — | — |  |
| HI | 1.19 | 1.01, 1.40 | 0.033 |
| **T Stage** |  |  |  |
| T1a | — | — |  |
| T1b | 3.70 | 1.67, 8.22 | 0.001 |
| T2 | 8.14 | 3.81, 17.4 | <0.001 |
| T3 | 17.4 | 8.07, 37.5 | <0.001 |
| T4 | 15.7 | 7.03, 35.1 | <0.001 |
| **N Stage** |  |  |  |
| N0 | — | — |  |
| N1 | 1.84 | 1.58, 2.13 | <0.001 |
| N2 | 2.98 | 2.40, 3.70 | <0.001 |
| Nx | 1.94 | 1.54, 2.44 | <0.001 |
| **Extent of Surgery** |  |  |  |
| CO | — | — |  |
| LLR | 1.05 | 0.81, 1.36 | 0.7 |
| MR | 1.19 | 0.91, 1.57 | 0.2 |
| **CCI Bracket** |  |  |  |
| <5 | — | — |  |
| >5 | 1.22 | 1.07, 1.40 | 0.003 |
| Abbreviations: CI = Confidence Interval, HR = Hazard Ratio | | | |

|  | **Optimal** | | | **Nearest** | | |
| --- | --- | --- | --- | --- | --- | --- |
| **Variable** | **HR** | **95% CI** | **p-value** | **HR** | **95% CI** | **p-value** |
| **age_boolean** | 1.12 | 0.95, 1.33 | 0.2 | 1.12 | 0.95, 1.32 | 0.2 |

Supplementary Figure 7: a) 90-day landmark analysis Cox regression on the unmatched and matched datasets on RFS

| **Variable** | **HR** | **95% CI** | **p-value** |
| --- | --- | --- | --- |
| **Age Bracket** |  |  |  |
| <75 | — | — |  |
| >=75 | 1.12 | 0.95, 1.32 | 0.2 |
| **sex** |  |  |  |
| Female | — | — |  |
| Male | 0.94 | 0.82, 1.07 | 0.4 |
| **R status** |  |  |  |
| R0 | — | — |  |
| R1 | 1.97 | 1.66, 2.34 | <0.001 |
| **Adjuvant Chemo** |  |  |  |
| No | — | — |  |
| Yes | 1.24 | 1.07, 1.43 | 0.004 |
| **Adjuvant radiotherapy** |  |  |  |
| No | — | — |  |
| Yes | 1.14 | 0.91, 1.43 | 0.2 |
| **Income Level** |  |  |  |
| NHI | — | — |  |
| HI | 1.25 | 1.06, 1.48 | 0.008 |
| **T Stage** |  |  |  |
| T1a | — | — |  |
| T1b | 3.60 | 1.61, 8.02 | 0.002 |
| T2 | 7.99 | 3.72, 17.2 | <0.001 |
| T3 | 16.9 | 7.79, 36.6 | <0.001 |
| T4 | 15.2 | 6.74, 34.1 | <0.001 |
| **N Stage** |  |  |  |
| N0 | — | — |  |
| N1 | 1.84 | 1.58, 2.15 | <0.001 |
| N2 | 3.03 | 2.42, 3.79 | <0.001 |
| Nx | 1.97 | 1.56, 2.50 | <0.001 |
| **Extent of Surgery** |  |  |  |
| CO | — | — |  |
| LLR | 1.04 | 0.79, 1.35 | 0.8 |
| MR | 1.10 | 0.83, 1.45 | 0.5 |
| **CCI Bracket** |  |  |  |
| <5 | — | — |  |
| >5 | 1.26 | 1.10, 1.45 | <0.001 |
| Abbreviations: CI = Confidence Interval, HR = Hazard Ratio | | | |

|  | **Optimal** | | | **Nearest** | | |
| --- | --- | --- | --- | --- | --- | --- |
| **Variable** | **HR** | **95% CI** | **p-value** | **HR** | **95% CI** | **p-value** |
| **age_boolean** | 1.10 | 0.93, 1.31 | 0.3 | 1.15 | 0.96, 1.37 | 0.13 |

Supplementary Figure 7: b) 120-day landmark analysis Cox regression on the unmatched and matched datasets on RFS

| **term** | **HR** | **95% CI** | **p-value** |
| --- | --- | --- | --- |
| **pspline(Age, df = 2), lin** | 1.00 | - | 0.797 |
| pspline(Age, df = 2), non | - | - | 0.010 |
| Sex Male | 0.89 | 0.79, 1.01 | 0.076 |
| R1 | 1.88 | 1.60, 2.20 | <0.001 |
| Adjuvant Chemo Yes | 0.99 | 0.86, 1.14 | 0.880 |
| Adjuvant RT Yes | 1.07 | 0.87, 1.32 | 0.499 |
| Income Level HI | 1.18 | 1.01, 1.39 | 0.038 |
| T1b | 3.36 | 1.58, 7.12 | 0.002 |
| T2 | 7.77 | 3.81, 15.84 | <0.001 |
| T3 | 18.40 | 8.95, 37.83 | <0.001 |
| T4 | 16.74 | 7.90, 35.48 | <0.001 |
| N1 | 1.88 | 1.63, 2.17 | <0.001 |
| N2 | 3.06 | 2.50, 3.74 | <0.001 |
| Nx | 1.96 | 1.58, 2.44 | <0.001 |
| Extent Surgery LLR | 1.06 | 0.83, 1.35 | 0.653 |
| Extent Surgery MR | 1.14 | 0.88, 1.48 | 0.314 |
| CCI Bracket>5 | 1.22 | 1.07, 1.40 | 0.004 |


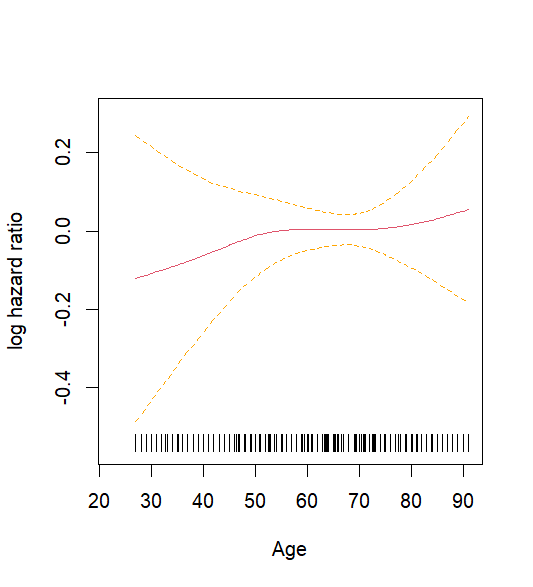


Supplementary Figure 8: Cox regression analysis for RFS using continuous age with splines

| **Variable** | **HR** | **95% CI** | **p-value** |
| --- | --- | --- | --- |
| **age70** |  |  |  |
| <70 | — | — |  |
| >70 | 0.99 | 0.86, 1.15 | 0.9 |
| **sex** |  |  |  |
| Female | — | — |  |
| Male | 0.90 | 0.79, 1.01 | 0.079 |
| **R status** |  |  |  |
| R0 | — | — |  |
| R1 | 1.87 | 1.60, 2.20 | <0.001 |
| **Adjuvant Chemo** |  |  |  |
| No | — | — |  |
| Yes | 0.98 | 0.86, 1.13 | 0.8 |
| **Adjuvant Radiotherapy** |  |  |  |
| No | — | — |  |
| Yes | 1.07 | 0.88, 1.32 | 0.5 |
| **Income Level** |  |  |  |
| NHI | — | — |  |
| HI | 1.20 | 1.03, 1.40 | 0.022 |
| **T Stage** |  |  |  |
| T1a | — | — |  |
| T1b | 3.37 | 1.59, 7.14 | 0.002 |
| T2 | 7.83 | 3.84, 16.0 | <0.001 |
| T3 | 18.6 | 9.03, 38.2 | <0.001 |
| T4 | 16.8 | 7.92, 35.6 | <0.001 |
| **N Stage** |  |  |  |
| N0 | — | — |  |
| N1 | 1.88 | 1.63, 2.17 | <0.001 |
| N2 | 3.06 | 2.50, 3.74 | <0.001 |
| Nx | 1.96 | 1.58, 2.44 | <0.001 |
| **Extent of Surgery** |  |  |  |
| CO | — | — |  |
| LLR | 1.05 | 0.82, 1.34 | 0.7 |
| MR | 1.13 | 0.88, 1.47 | 0.3 |
| **CCI Bracket** |  |  |  |
| <5 | — | — |  |
| >5 | 1.24 | 1.08, 1.42 | 0.002 |
| Abbreviations: CI = Confidence Interval, HR = Hazard Ratio | | | |

|  | **Optimal** | | | **Nearest** | | |
| --- | --- | --- | --- | --- | --- | --- |
| **Variable** | **HR** | **95% CI** | **p-value** | **HR** | **95% CI** | **p-value** |
| **age_boolean** | 1.03 | 0.91, 1.16 | 0.7 | 1.02 | 0.91, 1.15 | 0.7 |

Supplementary Figure 9a): Cox regression analysis of the matched and unmatched cohorts for RFS with cut-off of >70 years

| **Variable** | **HR** | **95% CI** | **p-value** |
| --- | --- | --- | --- |
| **age** |  |  |  |
| <80 | — | — |  |
| >80 | 1.14 | 0.92, 1.40 | 0.2 |
| **sex** |  |  |  |
| Female | — | — |  |
| Male | 0.90 | 0.79, 1.01 | 0.078 |
| **R status** |  |  |  |
| R0 | — | — |  |
| R1 | 1.88 | 1.60, 2.20 | <0.001 |
| **Adjuvant Chemo** |  |  |  |
| No | — | — |  |
| Yes | 1.00 | 0.87, 1.14 | >0.9 |
| **Adjuvant radiotherapy** |  |  |  |
| No | — | — |  |
| Yes | 1.08 | 0.88, 1.33 | 0.5 |
| **Income Level** |  |  |  |
| NHI | — | — |  |
| HI | 1.19 | 1.02, 1.38 | 0.025 |
| **T Stage** |  |  |  |
| T1a | — | — |  |
| T1b | 3.34 | 1.57, 7.07 | 0.002 |
| T2 | 7.70 | 3.78, 15.7 | <0.001 |
| T3 | 18.3 | 8.91, 37.6 | <0.001 |
| T4 | 16.6 | 7.83, 35.2 | <0.001 |
| **N Stage** |  |  |  |
| N0 | — | — |  |
| N1 | 1.88 | 1.63, 2.17 | <0.001 |
| N2 | 3.06 | 2.50, 3.74 | <0.001 |
| Nx | 1.94 | 1.57, 2.42 | <0.001 |
| **Extent of Surgery** |  |  |  |
| CO | — | — |  |
| LLR | 1.07 | 0.84, 1.37 | 0.6 |
| MR | 1.16 | 0.89, 1.50 | 0.3 |
| **CCI Bracket** |  |  |  |
| <5 | — | — |  |
| >5 | 1.22 | 1.08, 1.38 | 0.001 |
| Abbreviations: CI = Confidence Interval, HR = Hazard Ratio | | | |

|  | **Optimal** | | | **Nearest** | | |
| --- | --- | --- | --- | --- | --- | --- |
| **Variable** | **HR** | **95% CI** | **p-value** | **HR** | **95% CI** | **p-value** |
| **age_boolean** | 1.13 | 0.88, 1.43 | 0.3 | 1.13 | 0.90, 1.42 | 0.3 |

Supplementary Figure 9b): Cox regression analysis of the matched and unmatched cohorts for RFS with cut-off of >80 years

| **Variable** | **OR**^1^ | **95% CI**^1^ | **p-value** |
| --- | --- | --- | --- |
| **ageBracket** |  |  |  |
| <75 | — | — |  |
| >75 | 1.34 | 0.98, 1.81 | 0.061 |
| **sex** |  |  |  |
| Female | — | — |  |
| Male | 1.00 | 0.78, 1.29 | >0.9 |
| **finalRstatus** |  |  |  |
| R0 | — | — |  |
| R1 | 1.32 | 0.95, 1.81 | 0.10 |
| **adjuvantChemo** |  |  |  |
| No | — | — |  |
| Yes | 0.58 | 0.43, 0.76 | <0.001 |
| **adjuvantRT** |  |  |  |
| No | — | — |  |
| Yes | 0.88 | 0.50, 1.49 | 0.6 |
| **incomeLevel** |  |  |  |
| NHI | — | — |  |
| HI | 0.79 | 0.58, 1.09 | 0.15 |
| **tumourStage** |  |  |  |
| T1a | — | — |  |
| T1b | 0.72 | 0.18, 3.01 | 0.6 |
| T2 | 3.02 | 1.18, 10.3 | 0.041 |
| T3 | 12.0 | 4.63, 41.2 | <0.001 |
| T4 | 16.0 | 5.71, 57.9 | <0.001 |
| **nodalStage** |  |  |  |
| N0 | — | — |  |
| N1 | 1.79 | 1.33, 2.42 | <0.001 |
| N2 | 3.53 | 2.38, 5.23 | <0.001 |
| Nx | 2.22 | 1.36, 3.57 | 0.001 |
| **extentSurgery** |  |  |  |
| CO | — | — |  |
| LLR | 0.94 | 0.54, 1.66 | 0.8 |
| MR | 1.23 | 0.70, 2.21 | 0.5 |
| **ccmiBracket** |  |  |  |
| <5 | — | — |  |
| >5 | 1.26 | 0.96, 1.65 | 0.089 |
| ^1^OR = Odds Ratio, CI = Confidence Interval | | | |

Supplementary Figure 10: Multivariable logistic regression of factors associated with 1-year OS in the full cohort. CCMI – Charlson Comorbidity Index, CO – cholecystectomy only, LR – limited liver resection, MR – major resection

| **Variable** | **OR**^1^ | **95% CI**^1^ | **p-value** |
| --- | --- | --- | --- |
| **sex** |  |  |  |
| Female | — | — |  |
| Male | 0.95 | 0.63, 1.43 | 0.807 |
| **finalRstatus** |  |  |  |
| R0 | — | — |  |
| R1 | 2.90 | 1.76, 4.81 | <0.001 |
| **adjuvantChemo** |  |  |  |
| No | — | — |  |
| Yes | 0.51 | 0.29, 0.88 | 0.016 |
| **adjuvantRT** |  |  |  |
| No | — | — |  |
| Yes | 1.21 | 0.38, 3.81 | 0.749 |
| **incomeLevel** |  |  |  |
| NHI | — | — |  |
| HI | 0.47 | 0.23, 0.94 | 0.034 |
| **tumourStage** |  |  |  |
| T1a | — | — |  |
| T1b | 1.86 | 0.36, 9.59 | 0.456 |
| T2 | 2.26 | 0.51, 10.12 | 0.286 |
| T3 | 6.87 | 1.45, 31.62 | 0.013 |
| T4 | 8.69 | 1.50, 50.19 | 0.016 |
| **nodalStage** |  |  |  |
| N0 | — | — |  |
| N1 | 2.63 | 1.64, 4.33 | <0.001 |
| N2 | 5.05 | 2.18, 10.52 | <0.001 |
| Nx | 2.48 | 1.30, 4.73 | 0.006 |
| **extentSurgery** |  |  |  |
| CO | — | — |  |
| LLR | 1.28 | 0.65, 2.50 | 0.476 |
| MR | 1.21 | 0.61, 2.49 | 0.596 |
| **ccmiBracket** |  |  |  |
| <5 | — | — |  |
| >5 | 1.17 | 0.75, 1.82 | 0.482 |
| ^1^OR = Odds Ratio, CI = Confidence Interval | | | |

Supplementary Figure 11: Multivariable logistic regression of factors associated with 1-year OS in the >75 year cohort. CCMI – Charlson Comorbidity Index, CO – cholecystectomy only, LR – limited liver resection, MR – major resection

| **Variable** | **HR** | **95% CI** | **p-value** |
| --- | --- | --- | --- |
| **sex** |  |  |  |
| Female | — | — |  |
| Male | 0.95 | 0.73, 1.25 | 0.7 |
| **R status** |  |  |  |
| R0 | — | — |  |
| R1 | 1.87 | 1.29, 2.72 | 0.001 |
| **Adjuvant Chemo** |  |  |  |
| No | — | — |  |
| Yes | 0.88 | 0.62, 1.25 | 0.5 |
| **Adjuvant radiotherapy** |  |  |  |
| No | — | — |  |
| Yes | 0.97 | 0.43, 2.17 | >0.9 |
| **Income Level** |  |  |  |
| NHI | — | — |  |
| HI | 0.85 | 0.50, 1.46 | 0.6 |
| **T Stage** |  |  |  |
| T1a | — | — |  |
| T1b | 1.21 | 0.46, 3.16 | 0.7 |
| T2 | 3.15 | 1.45, 6.85 | 0.004 |
| T3 | 7.49 | 3.28, 17.1 | <0.001 |
| T4 | 5.96 | 2.09, 17.0 | <0.001 |
| **N Stage** |  |  |  |
| N0 | — | — |  |
| N1 | 2.15 | 1.55, 2.99 | <0.001 |
| N2 | 3.18 | 1.76, 5.72 | <0.001 |
| Nx | 2.15 | 1.42, 3.27 | <0.001 |
| **Extent of Surgery** |  |  |  |
| CO | — | — |  |
| LLR | 0.97 | 0.63, 1.50 | 0.9 |
| MR | 0.87 | 0.54, 1.42 | 0.6 |
| **CCI Bracket** |  |  |  |
| <5 | — | — |  |
| >5 | 0.87 | 0.66, 1.14 | 0.3 |
| Abbreviations: CI = Confidence Interval, HR = Hazard Ratio | | | |

Supplementary Figure 12:a) 90-day landmark analysis Cox regression on OS in the >75 year cohort

| **Variable** | **HR** | **95% CI** | **p-value** |
| --- | --- | --- | --- |
| **sex** |  |  |  |
| Female | — | — |  |
| Male | 0.97 | 0.73, 1.27 | 0.8 |
| **R status** |  |  |  |
| R0 | — | — |  |
| R1 | 1.68 | 1.14, 2.49 | 0.009 |
| **Adjuvant Chemo** |  |  |  |
| No | — | — |  |
| Yes | 0.88 | 0.61, 1.27 | 0.5 |
| **Adjuvant radiotherapy** |  |  |  |
| No | — | — |  |
| Yes | 1.03 | 0.45, 2.38 | >0.9 |
| **Income Level** |  |  |  |
| NHI | — | — |  |
| HI | 0.82 | 0.46, 1.45 | 0.5 |
| **T Stage** |  |  |  |
| T1a | — | — |  |
| T1b | 1.23 | 0.47, 3.23 | 0.7 |
| T2 | 3.18 | 1.45, 6.98 | 0.004 |
| T3 | 7.47 | 3.23, 17.3 | <0.001 |
| T4 | 5.92 | 2.04, 17.1 | 0.001 |
| **N Stage** |  |  |  |
| N0 | — | — |  |
| N1 | 2.10 | 1.49, 2.94 | <0.001 |
| N2 | 3.48 | 1.90, 6.38 | <0.001 |
| Nx | 2.20 | 1.43, 3.39 | <0.001 |
| **Extent of Surgery** |  |  |  |
| CO | — | — |  |
| LLR | 0.94 | 0.60, 1.48 | 0.8 |
| MR | 0.85 | 0.51, 1.40 | 0.5 |
| **CCI Bracket** |  |  |  |
| <5 | — | — |  |
| >5 | 0.87 | 0.66, 1.14 | 0.3 |
| Abbreviations: CI = Confidence Interval, HR = Hazard Ratio | | | |

Supplementary Figure 12:b) 120-day landmark analysis Cox regression on OS in the >75 year cohort

| **Variable** | **OR**^1^ | **95% CI**^1^ | **p-value** |
| --- | --- | --- | --- |
| **sex** |  |  |  |
| Female | — | — |  |
| Male | 1.10 | 0.77, 1.57 | 0.614 |
| **incomeLevel** |  |  |  |
| NHI | — | — |  |
| HI | 1.14 | 0.59, 2.21 | 0.691 |
| **extentSurgery** |  |  |  |
| CO | — | — |  |
| LLR | 0.94 | 0.57, 1.56 | 0.814 |
| MR | 3.09 | 1.86, 5.14 | <0.001 |
| **ccmiBracket** |  |  |  |
| <5 | — | — |  |
| >5 | 1.45 | 0.98, 2.14 | 0.063 |
| ^1^OR = Odds Ratio, CI = Confidence Interval | | | |

Supplementary Figure 13: Multivariable logistic regression of factors associated with complications in the >75 year cohort. CCMI – Charlson Comorbidity Index, CO – cholecystectomy only, LR – limited liver resection, MR – major resection

| **Cause of death** | **Overall cohort** | **<75 years** | **>75 years** |
| --- | --- | --- | --- |
| Liver failure | 29 | 27 | 2 |
| Biliary sepsis | 27 | 14 | 13 |
| Bleeding | 19 | 15 | 4 |
| Respiratory failure | 8 | 7 | 1 |
| Cardiovascular | 8 | 4 | 4 |
| Pancreatic leak | 6 | 6 | 0 |
| Pulmonary embolism | 5 | 3 | 2 |
| Renal failure | 3 | 2 | 1 |
| Early progression | 2 | 0 | 2 |
| Unknown | 15 | 9 | 6 |
| Total | 122 | 87 | 35 |

**Supplementary Table 2: Causes of 90-day mortality in older and younger adults**
